# Using fuzzy logic to compare species distribution models developed on the basis of expert knowledge and sampling records: Expert knowledge versus sampling in species distribution modelling

**DOI:** 10.1186/s12983-023-00515-x

**Published:** 2023-12-07

**Authors:** David Romero, Raúl Maneyro, José Carlos Guerrero, Raimundo Real

**Affiliations:** 1 Biogeography, Diversity, and Conservation Research Team, Department of Animal Biology, Faculty of Sciences, Universidad de Málaga, Málaga, Spain; 2 Laboratory of Systematics and Natural History of Vertebrates, Faculty of Sciences, Universidad de La República, Montevideo, Uruguay; 3 Laboratory for Sustainable Development and Environmental Management, Faculty of Sciences, Universidad de La República, Montevideo, Uruguay

**Keywords:** Amphibians, Incomplete records, Favourable areas, Fuzzy consensus, Non-observed species, Potential biodiversity, Threatened species

## Abstract

**Background:**

Experts use knowledge to infer the distribution of species based on fuzzy logical assumptions about the relationship between species and the environment. Thus, expert knowledge is amenable to fuzzy logic modelling, which give to propositions a continuous truth value between 0 and 1. In species distribution modelling, fuzzy logic may also be used to model, from a number of records, the degree to which conditions are favourable to the occurrence of a species. Therefore, fuzzy logic operations can be used to compare and combine models based on expert knowledge and species records. Here, we applied fuzzy logic modelling to the distribution of amphibians in Uruguay as inferred from expert knowledge and from observed records to infer favourable locations, with favourability being the commensurable unit for both kinds of data sources. We compared the results for threatened species, species considered by experts to be ubiquitous, and non-threatened, non-ubiquitous species. We calculated the fuzzy intersection of models based on both knowledge sources to obtain a unified prediction of favourable locations.

**Results:**

Models based on expert knowledge involved a larger number of variables and were less affected by sampling bias. Models based on experts had the same overprediction rate for the three types of species, whereas models based on species records had a lower prediction rate for ubiquitous species. Models based on expert knowledge performed equally as well or better than corresponding models based on species records for threatened species, even when they had to discriminate and classify the same set of records used to build the models based on species records. For threatened species, expert models predicted more restrictive favourable territories than those predicted based on records. Observed records generated the best-fitted models for non-threatened non-ubiquitous species, and ubiquitous species.

**Conclusions:**

Fuzzy modelling permitted the objective comparison of the potential of expert knowledge and incomplete distribution records to infer the territories favourable for different species. Distribution of threatened species was able to be better explained by subjective expert knowledge, while for generalist species models based on observed data were more accurate. These results have implications for the correct use of expert knowledge in conservation planning.

**Supplementary Information:**

The online version contains supplementary material available at 10.1186/s12983-023-00515-x.

## Background

The status of species presence in a territory is usually subject to doubt [1]. This is mainly due to poor conducted sampling and to the dynamic nature of species distributions [2]. Imprecise knowledge of ranges occupied by species can lead to erroneous inferences about the species-environment relationship [3], which may have a long-term impact on biodiversity conservation [4]. However, at least part of the response of species to environmental conditions can be analysed based on the locations where they have been recorded; such results may then be used to infer the possible distribution of species in other locations with similar conditions [5, 6].

Species distribution modelling has been used to objectively evaluate the potential of areas to be occupied by species [5, 7]. This potential is typically established according to the species-environment relationship, which can be inferred from known species occurrence data [2, 5, 8]. Experts on a species in a particular territory also tend to use their expertise to subjectively infer the wider distribution of species in a territory, typically at the country or regional level. Experts’ experience and knowledge of the biology of species are used to define and review the distribution of a species, thus improving the available information [9]. Expert-derived distribution maps may be compared and combined with distribution models based on recorded data to improve model outputs, although procedures through which to do this have only recently begun to be explored [9–11].

In Uruguay, recorded species distribution data, although generally incomplete, is mainly available in scientific collections [12, 13], scientific publications [14–19], in the Global Biodiversity Information Facility [20], or in the Biodiversidata database [13, 21]. Amphibians are one of the taxa that remain poorly characterized in Uruguay [18] and are one of the most threatened groups on the planet [22, 23]. It is challenging to perform effective sampling of amphibians because of their two-phase life cycle, both phases of which are affected to varying degrees by local environmental fluctuations. Many of these species also have a crepuscular lifestyle, have cryptic colour patterns, live underground, or can only be found during specific seasons or under particular weather conditions [24]. Maneyro & Carreira [17] published a field guide to Uruguayan amphibians in which they presented the areas they considered to be occupied by each species according to their expert knowledge. These expert-defined areas are implicitly derived from fuzzy hypotheses about the relationship between species and the environment inferred from direct observation of species in their habitats. Thus, fuzzy logic may be appropriate for formally analysing these inferences.

Fuzzy logic was introduced by Zadeh [25] as a many-valued logic in which propositions have a truth value that varies in degree from 0 (completely false) to 1 (completely true). This logic is particularly useful for formally analysing the kinds of messages involved in human language and thinking [26]. Consequently, fuzzy logic may be used to make experts' assumptions more formally explicit, which could help to more objectively define environmental conditions that are considered as favourable for a species according to expert knowledge. Fuzzy logic may also be used to objectively model on the basis of an incomplete number of records, the degree to which conditions are favourable for the presence of a species in a territory in which the species has not yet been found [5, 27]. Specifically, favourability is a mathematical function that estimates, based on the probability of occurrence and species prevalence, the degree to which environmental conditions are favourable for a species’ occurrence in an area. With these fuzzy favourability values, it is possible to perform fuzzy logic operations that can be used account for the uncertainty inherent in nature, in which conditions are only ever partially favourable for the occurrence of a species, into species distribution models [6, 8, 28].

The application of fuzzy logic modelling to experts’ inferences and to occurrence records places both kinds of data within the same conceptual framework. It also yields commensurable degrees of affiliation with a fuzzy set of locations with favourable conditions for a species according to the two datasets. In addition, fuzzy logic operations may be used to compare and combine both kinds of models once they are expressed in commensurable favourability units.

The aim of this study was to test the capability of fuzzy logic tools to compare and combine the distribution of species inferred from expert criteria and from incomplete records of species distribution. This may have implications for the better use of expert knowledge in conservation planning and for identifying the most favourable conditions and territories for species.

## Materials and methods

### Study area and species distribution data

**Fig. 1:**
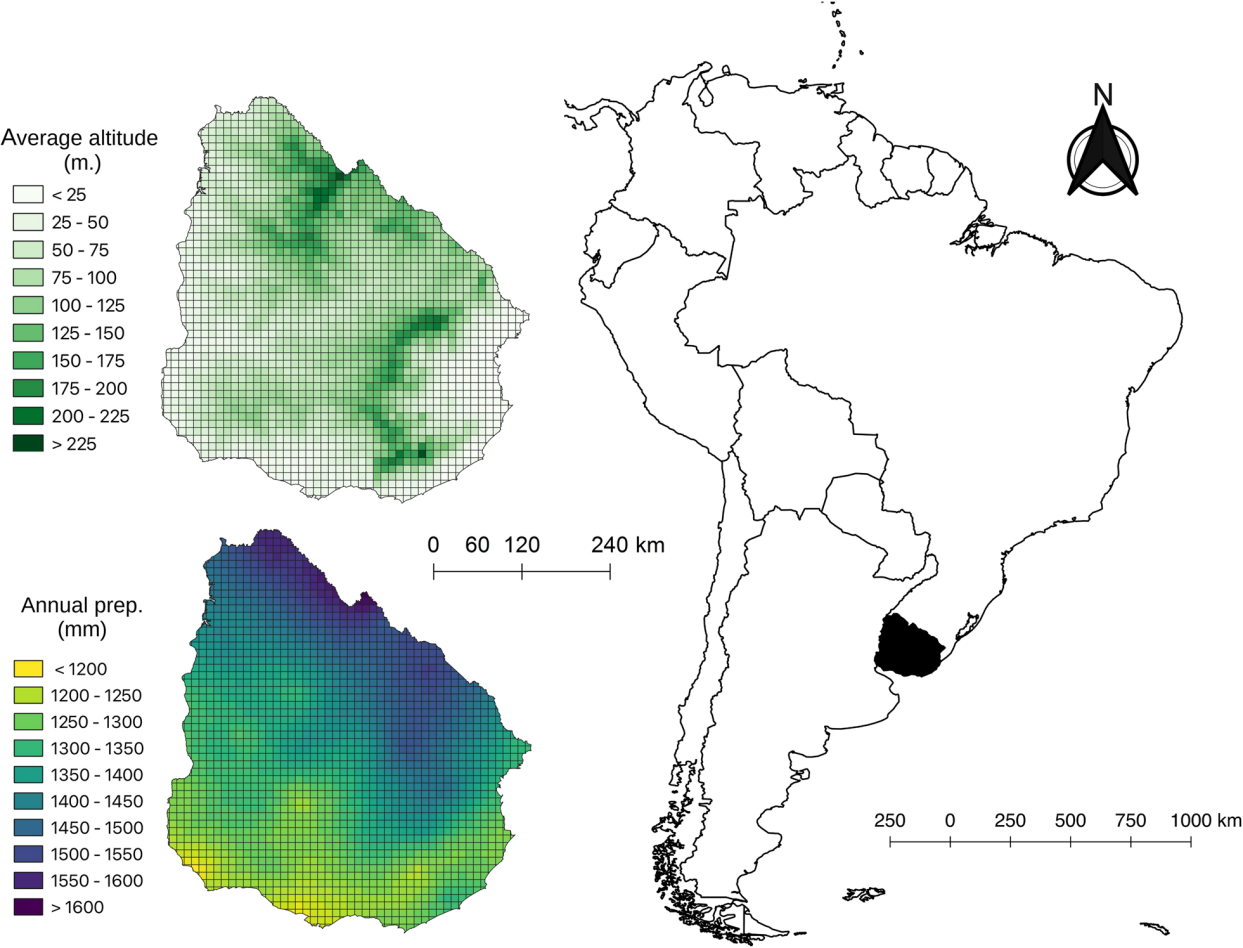
10-km × 10-km grid cells of Uruguay and situation of Uruguay within South America. As examples, the display of the values of two explanatory variables on this grid is shown

This study was carried out in Uruguay, which is located between 30° to 35° south latitude and 53° to 55° west longitude, lies entirely within the temperate zone and, according to Olson et al. [29], is included in the Uruguayan savanna ecoregion. It has low orography, with average altitudes not exceeding 514 m above sea level, and a wide coastline on the Atlantic Ocean (more than 150 km; see average altitude map in Fig. 1). The proximity to the sea regulates the average annual temperature (approximately 17.5 °C) and precipitation varies annually between 1000 and 1700 mm [30] (see annual precipitation map in Fig. 1). Uruguay is located at a biogeographic crossroads created by the influence of various biotas [31].

The country has high amphibian species richness, with 48 native species (one gymnophion, 47 anurans), and an invasive exotic anuran (*Lithobates catesbeianus*). As in other regions of the world, amphibians are in decline [22, 23], with 12 species under some category of threat [18] (Table S1 in Additional file 1) and seven considered as almost threatened, not evaluated, or with insufficient data [18, 32]. Recently, amphibian species nomenclature was revised and updated (see [33] for details).

To compare species records and maps derived from expert knowledge, an operational scale of resolution capable of reconciling the different precisions of species records and expert distribution maps must be used. The spatial precision of expert maps is usually unknown [34, 35], but generally considered to be broad, in the range of 100–200 km [36]. Species distribution records have a higher precision, but a common intermediate precision scale to assess the two types of data in a commensurate had to be defined. We used a grid divided Uruguay country into 10 km × 10 km cells (total: 1887 grid cells; Fig. 1). This is in line with methods used in biogeography to identify the best grid for biogeographical studies [37]. We verified the recorded or expert-inferred presence of each amphibian species in each grid cell. To do this, we reviewed two types of information sources: distribution data recorded in the databases of scientific collections, and expert criteria, understood as the distribution maps derived from expert knowledge of the species' natural history. Regarding the recorded distribution, information on the geo-referenced occurrences of the species was extracted from the amphibian collection database of the Faculty of Sciences of the University of the Republic of Uruguay [21] and from the work of Núñez et al. [12]. Thus, grid cells with at least one record were considered as indicating species presence and the rest as absence. Distribution according to expert criteria were taken from the guide to amphibians in Uruguay [17]. In this manner, we considered the grid cells totally or partially contained in the range established by the experts for each species as grid cells with presence of the species. The remaining grid cells were treated as absences. We divided species into three categories to compare how models based on species records or expert knowledge performed in each category: threatened species (hereafter T), species considered by experts to be present throughout the study area (ubiquitous species, hereafter U), and non-threatened, non-ubiquitous species (hereafter NtNu).

### Environmental predictor variables

For predictors, we used a set of 44 variables linked to spatial location (combination of longitude and latitude) and to the following eight environmental factors: topography (four variables), climate (22 variables), vegetation (one variable), geography (one variable), hydrology (two variables), land cover (five variables), lithology (four variables) and human activity (four variables). These are the factors that, according to theory, more likely affect the species analysed at the spatial resolution of our study [38]. Table 1 shows the details of variables and their sources. Presence/absence of each amphibian species and the values of the predictors variables were extracted for each 10-km × 10-km grid cell and stored in a geo-referenced database implemented in a Geographical Information System using QGIS software [39].

**Table 1:** Explanatory variables analysed during the modelling process and their source. Variable names (second and forth columns), their codes (first and third columns) and factors grouping them (inserted sections). The resolution of the variables when coming from a raster are indicated in brackets next to the factor in which are grouped, in italics. The codes of the variables selected after the multicollinearity evaluation are shown in bold

Code	Variables	Code	Variables
Spatial			
**YSp**	Spatial logit (linear polynomial combination of Latitude (°N) and Longitude (°E) from the spatial logistic regression)^(1)^		
Topography *(1 km* × *1 km of original resolution)*			
**A**	Average altitude (m)^(2)^	**S**	Slope (◦) (calculated from Altitude)
**Ori-NS**	Orientation; degrees of exposure NS (calculated from Slope)	**Ori-EW**	Orientation; degrees of exposure EW (calculated from Slope)
Climatic *(1 km* × *1 km of original resolution)*			
**BIO**_**1**_	Average annual temperature (°C)^(3)^	BIO_11_	Mean annual temperatures of the coldest quarter (°C)^(3)^
BIO_2_	Mean diurnal range temperatures (°C)^(3)^	**BIO**_**12**_	Annual precipitation (mm)^(3)^
BIO_3_	Isothermality (BIO_2_/BIO_17_) (*100) (°C)^(3)^	BIO_13_	Precipitation of the wettest month (mm)^(3)^
BIO_4_	Seasonality of temperatures (°C)^(3)^	BIO_14_	Precipitation of the driest month (mm)^(3)^
BIO_5_	Maximum temperatures of the warmest month (°C)^(3)^	**BIO**_**15**_	Seasonality of precipitation (mm)^(3)^
BIO_6_	Minimum temperatures of the coldest month (°C)^(3)^	BIO_16_	Precipitation of wettest quarter (mm)^(3)^
** BIO**_**7**_	Annual temperature range (BIO_5_-BIO_6_)^(3)^	BIO_17_	Precipitation of dry quarter^(3)^
BIO_8_	Mean annual temperatures of the wetter quarter^(3)^	**BIO**_**18**_	Precipitation of warmest quarter^(3)^
BIO_9_	Mean annual temperatures of the dry quarter^(3)^	**BIO**_**19**_	Precipitation of coldest quarter^(3)^
BIO_10_	Mean annual temperatures of the warmest quarter^(3)^	**PMax**	Maximum average precipitation in 24 h (mm)^(3)^
** BhPri**	Spring water balance (mm)^(3)^	**ETR**	Monthly real evapotranspiration (mm)^(3)^
Vegetation *(1 km* × *1 km of original resolution)*			
** NDVI**	Index of greenness^(4)^		
Geography			
** DistCost**	Distance to coast (km)^(5)^		
Hydrology			
** DistRiver**	Minimum distance to rivers (km)^(6)^	*LonRiver*	Longitude of rivers (km)^(6)^
Land use			
**Forests**	Forests (%)^(7)^	**Reforests**	Reforestation (%)^(7)^
**NatField**	Natural field (%)^(7)^	**Crops**	Crops (%)^(7)^
**Wetland**	Wetland (%)^(7)^		
Lithology			
**DepthSoil**	Depth of soil^(8)^	**TextSoil**	Soil texture^(8)^
**RockySoil**	Rocky soil^(8)^	**FloodSoil**	Flood soil^(8)^
Human activities *(1 km* × *1 km of original resolution)*			
**PobDen**	Population density^(9)^	**DistUrban**	Minimum distance to the main urban centers (Km)^(10)^
**DistRoad**	Minimum distance to paved roads (km)^(11)^	**DistUnpavRoad**	Distance to unpaved roads (km)^(11)^

Sources:

(1) Spatial variables, latitude, and longitude, were generated using the vector geometry tools of QGIS (http://www.qgis.org) software: (a) "centroids of polygons" was used to calculate the centroid of each grid cell was calculated; and (b) "Export/Add columns of geometry" was used to express the length and latitude values (1984 World Geodetic System) assigned to each centroid (WGS84)

(2) United States Geological Survey (1996). GTOPO30. Land Processes Distributed Active Archive Center. EROS Data Center: https://lta.cr.usgs.gov/GTOPO30. (accessed April 2016)

(3) Ceroni (2008) from DNM-INIA. Monthly data series for 30 years for Uruguay (from 1980 to 2009). We calculated the bioclimatic variables (BIO1–BIO19) following the proposal used in WorldClim (Fick & Hijmans, 2017)

(4) https://www.vito-eodata.be: from SPOT-VEGETATION – S10 NDVI

(5) Generated using QGIS (http://www.qgis.org) software by calculating the average distance from the centroid of the grid cell to the coastline

(6) United States Geological Survey (2006). HydroShed. Hydrological data and maps based on SHuttle Elevation Derivatives at multiple Scales. Available at: http://hydrosheds.cr.usgs.gov/index.php/ (accessed May 2016)

(7) GlobCover (2009). Global land cover map. Available at: http://due.esrin.esa.int/page_globcover.php (accessed April 2016)

(8) Panario & Gutiérrez (2011). Mapa de ambientes: Cartografía implementada en un SIG. In: Mapa de Ambientes de Uruguay y Distribución potencial de especies, Convenio MGAP/PPR-CIEDUR, Montevideo

(9) Gridded Population of the World (GPWv4) (2010). Socioeconomic Data and Applications Center (SEDAC). A Data Center in NASA's Earth Observing System Data and Information System (EOSDIS)—Hosted by CIESIN at Columbia University (accessed June 2016)

(10) Natural Earth Data. North American Cartographic Information Society (NACIS). Available at: http://www.naturalearthdata.com/ (accessed April 2016)

(11) Digital Chart of the World. Available at: https://worldmap.harvard.edu/data/geonode:Digital_Chart_of_the_World (accessed April 2016)

Regarding the spatial structure of the distribution data, a polynomial trend-surface analysis [40, 41] was applied using the longitude (Lo), latitude (La), the quadratic and cubic effect of both, and a combination of the above (Lo; La; Lo2; Lo3; La2; La3; LaLo; La2Lo; LaLo2). The spatial variable included in the models for each species was a linear multifactorial combination (hereafter spatial logit) resulting from a linear logistic regression of the species presence/absence on the combination of these nine spatial terms of latitude and longitude. This spatial analysis determined whether the distribution of a species responded to spatial geographic trends related to historical events or more recent population dynamics, rather than to the environmental conditions of the habitat they occupy [41, 42]. For the other variables, we avoided the use of Principal Component Analysis or quadratic terms to keep the models simple and facilitate comparison with models based on expert knowledge, as such models are usually based on a simpler relationship between the species distribution and environmental conditions.

### Favourability models

To avoid excessive multicollinearity in the models, we tested pair-wise correlations between non-spatial variables using the Spearman coefficient r. When two variables had a correlation value of r > 0.75, we only retained the variable that the expert considered more relevant regarding amphibians, as this option facilitated the comparison of the explanatory capacity of models based on expert knowledge and on species records. This procedure reduced the number of potential predictor variables to 30 (Table 1). For each species, we then performed bivariate logistic regression models of the species distribution with each of these variables to obtain a yet smaller subset of significant predictors, according to the Rao’s score test, for each species. If no predictor had a significant relationship with the distribution of the species, then no model was produced for the species and source of data.

To minimize the risk of Type I errors in the modelling process due to the number of predictors, we also controlled for the false discovery rate (FDR) [43] for each species, retaining only those predictor variables that were significant under an FDR of q < 0.05.

Then, we performed an ensemble multivariate forward–backward stepwise logistic regression of presences/absences of each species on their corresponding spatial variable and reduced set of environmental variables to obtain probability values (P) of the species’ presence in every grid cell according to the formula:

$${\text{P}} = {\text{e}}^{{\text{y}}} /\left( {{1} + {\text{ e}}^{{\text{y}}} } \right),$$

with e being the base of the natural logarithms and y the logit function

$${\text{y}} = \alpha + \beta_{{1}} {\text{x}}_{{1}} + \beta_{{2}} {\text{x}}_{{2}} + \beta_{{3}} {\text{x}}_{{3}} + \cdots + \beta_{{\text{n}}} {\text{x}}_{{\text{n}}}$$

where, α is a constant and β_1,_ β_2,…,_ β_n_ are the coefficients of the n environmental predictor variables x_1,_ x_2,…,_ x_n_ (see details in Acevedo & Real [5] and Real et al. [27]). Variables were included according to the importance of their relationship with species distribution, and redundancy was avoided by verifying at each step that each new variable added significant new information.

Subsequently, favourability values were calculated from the probability values obtained by logistic regression according to the formula:

$${\text{F}} = \left[ {{\text{P}}/\left( {{1} - {\text{P}}} \right)} \right]/\left[ {\left( {{\text{n1}}/{\text{n}}0} \right) + ({\text{P}}/} \right[{1} - {\text{P}}\left] ) \right]$$

where F is the environmental favourability (between 0 and 1), P is the probability of occurrence given by the multivariate logistic regression, n1 is the number of presences, and n0 is the number of absences [27]. Thus, when the number of presences and absences is the same, F = P, whereas when the number of absences is greater than the number of presences, F > P, and when the number of absences is lower than presences F < P (which is rarely the case). The favourability value F = 0.5 corresponds to a probability value equal to the prevalence of the species in the sample, i.e., the value expected under neutral conditions over the entire area of study. Thus, the favourability values represent the contribution of the response of the species to the local environmental conditions and of its particular history and population dynamics to the probability of occurrence. It should be noted that local probability depends on local favourability and on the overall prevalence of species in the dataset. Therefore, favourability is a commensurate unit that can be used to compare and combine models of species that differ in their prevalence [44, 45].

A favourability function can be considered to be a membership function of each locality in the fuzzy set of localities favourable for the occurrence of species. This matches the definition of the membership function typical of fuzzy sets, which is the basis of fuzzy logic [25]. This logic asserts that the membership of any element in a set is neither completely false nor completely true; instead, each element is assigned a real number from 0 to 1 representing the degree of membership in the fuzzy set. After applying the favourability function, each grid cell corresponds to a degree of membership, ranging from 0 to 1, to the set of favourable localities for the presence of each amphibian species. For each grid cell, the favourability function can identify dark biodiversity or the degree of favourability for non-observed species [6]. It can also combine favourability models using fuzzy logic operations [44–47].

The above-mentioned procedure was applied to the presence/absence datasets derived from the species ranges proposed by experts and to those derived from recorded distributions. In this manner, favourability values were obtained based on expert knowledge (F_E_) and recorded distributions (F_R_).

All statistical analyses were conducted using the IBM SPSS Statistics V25 software. Finally, a cartographic favourability model was generated for each amphibian species according to both sources of data using ArcMap 10.8 software.

### Model evaluation

We used the area under the curve (AUC) of the receiving operating characteristic (ROC) [48, 49] to evaluate the ability of each favourability model, either based on species records or on expert knowledge, to discriminate grid cells with at least one record from those with no record of the species. We evaluated the ability of each model to classify this dataset of recorded presences and absences based on a favourability threshold of 0.5 using sensitivity, specificity, under-prediction rate (UPR), over-prediction rate (OPR) [50] and correct classification rate (CCR) [51]. We also compared the discrimination and classification ability of the models for T species (N = 12), NtNu (N = 22), and U (N = 14), using the Kruskal–Wallis Test to separately test models based on species records and on expert knowledge. We also compared the number of variables and factors involved in the models, as well as the discrimination and classification ability of models based on species records and expert criteria using the Wilcoxon signed-rank test for matched samples for the three groups of species separately.

Favourability values (F) represent the degree of membership in the fuzzy set of grid cells favourable for the occurrence of the species. Consequently, we obtained for each species a fuzzy set of grid cells favourable for the occurrence of the species according to expert criteria (E) with favourability values F_E,_ and another set according to the direct modelling of the recorded distribution data (R) with favourability values F_R_. For each of these fuzzy sets, we computed cardinal, card (E) and card (R), which is the sum of all favourability values representing the size of the fuzzy set. We then computed the fuzzy entropy of each fuzzy set [26] as follows:

$$\begin{gathered} {\text{S}}\left( {\text{E}} \right) \, = {\text{ card }}({\text{E}} \cap {\text{E}}^\prime /{\text{card }}({\text{E}} \cup {\text{E}}^\prime ) \hfill \\ {\text{S}}\left( {\text{R}} \right) \, = {\text{ card }}\left( {{\text{R}} \cap {\text{R}}^\prime } \right)/{\text{card }}({\text{R}} \cup {\text{R}}^\prime ) \hfill \\ \end{gathered}$$

where S(E) is the entropy of E, S(R) is the entropy of R, and E′ and R′ are the complementary values of E and R, respectively, whose membership values in each grid cell are 1-F_E_ and 1-F_R_, respectively. Fuzzy entropy is a value between 0 (minimum entropy) and 1 (maximum entropy) that represents the uncertainty associated with the species distribution. Using the Wilcoxon signed-rank test for matched samples, we compared the fuzzy entropy of models based on species records and expert criteria for the three groups of species separately.

### Combining fuzzy sets derived from records and expert criteria using fuzzy logic

These two fuzzy sets were combined using fuzzy logic tools [25]. Then, for each species, we calculated in each grid cell the degree of membership in the fuzzy union E ∪ R (maximum of F_E_ or F_R_) and the fuzzy intersection E ∩ R (minimum of F_E_ and F_R_) of the two fuzzy sets as the consensus values of both models. E ∪ R represents the degree to which a location is considered favourable according to either expert criteria or the direct modelling of the records, whereas E ∩ R represents the degree to which a location is considered favourable using both expert criteria and direct modelling of the records. E ∪ R and E ∩ R were also fuzzy sets and we represented them cartographically and as computed card (E ∪ R) and card (E ∩ R).

We also obtained the fuzzy overlap values between E and R, which were values between 0 and 1 computed as:

$${\text{O }}\left( {{\text{E}},{\text{R}}} \right) \, = {\text{ card }}\left( {{\text{E}} \cap {\text{R}}} \right)/{\text{card }}({\text{E}} \cup {\text{R}})$$

To assess how much information R held about E, we used the fuzzy inclusion of E into R according to the equation provided by [52]:

$${\text{I }}\left( {{\text{E}},{\text{ R}}} \right) \, = {\text{ card }}\left( {{\text{E}} \cap {\text{R}}} \right)|/{\text{card }}\left( {\text{E}} \right)$$

which is a value between 1 and 0 that indicates the extent to which the set E is included in the set R. In this way, we computed I (R, E).

In all methodological procedures, we followed the recommendations of Sillero et al. [53].

## Results

After accounting for multicollinearity among the original variables and controlling for FDR, a total of thirty predictor variables belonging to the nine explanatory factors remained available for building the models. We did not obtain significant favourability models for species with ubiquitous distributions using expert inference, as the experts considered that these distributions covered all of Uruguay. We obtained significant favourability models for the rest of the included species using both expert criteria and recorded distributions (see the geographical representation of the models in maps of Figure S3 in Additional file 3).

### Explanatory factor and variables in the models

The thirty variables available to build the models, and the nine consequent factors, were included in at least one significant distribution model. Relevant variables differed according to the two sources of information analysed. The expert-criteria-based models were significantly more complex, as they included between one and 16 variables, with a mean of 5.1 and 8.8 variables per model for threatened and NtNu species, respectively, whereas the models based on recorded distributions included between one and eight variables, with a mean of 1.9 and 2.7 variables per model for T and NtNu species, respectively (Wilcoxon signed-rank test significance was p < 0.05 for T species and p < 0.001 for NtNu species), and 5.8 variables per model for U species. Consequently, expert-criteria-based models included more explanatory factors than record-based models.

According to the expert criteria, the three most representative factors in the models were climatic (85% of models), topographic (74% of models) and spatial (62% of models), whereas according to the recorded distribution, the most representative factors were spatial (54% of models), land use (54% of models) and climatic (52% of models).

In models based on the recorded distributions, distances to roads and urban centres were included 24 times, and only once with a positive value, whereas in models based on expert criteria, these variables were included 20 times, 10 which had positive values (χ^2^ = 9.147, p < 0.01).

### Discrimination capacity of the models

In general, the models were able to discriminate presences from absences (see mean values of AUC in Table 2). Using both sources of data, all models of the 12 threatened species showed outstanding discrimination according to Hosmer & Lemeshow [48] (AUC > 0.9). Wilcoxon signed-rank test results showed no significant differences between them (Table 3), as both sources of data performed equally well. However, some outlier models had lower discrimination in models based on species records, meaning that models based on expert knowledge discriminated equally as well as or better than corresponding models based on species records for T species (Figure S1 in Additional file 2). In fact, the Kruskal–Wallis test showed that three significantly different levels of discrimination ability could be distinguished for models based on expert knowledge, while only two levels could be distinguished from models based on species records (one for ubiquitous species and one for the rest of species) (see Table 4, and Figure S1 in Additional file 2).

**Table 2:** Number of species (N), average discrimination (AUC or Area Under the Curve), and classification values (Sensitivity, Specificity, CCR or Correct Classification Rate, Und or Under Prediction Rate and Ove or Over Prediction Rate) for all species, and separately for Threatened species (T), non-threatened non-ubiquitous species (NtNu), and ubiquitous species (U) of the models based on expert criteria and on species records

According to experts							
Species	N	AUC	Sensitivity	Specificity	CCR	Und	Ove
T	12	0.98 (0.961–0.995)	0.97 (0.857–0.999)	0.94 (0.845–0.969)	0.94 (0.845–0.969)	0.00028 (0–0.00168)	0.90 (0.657–0.986)
NtNu	22	0.82 (0.475–0.999)	0.87 (0.361–0.999)	0.68 (0.211–0.998)	0.69 (0.24–0.998)	0.0044 (0–0.0266)	0.93 (0.636–0.992)
U	14	0.5 (0.5–0.5)	1 (0.999–0.999)	0 (0–0)	0.083 (0.0472–0.152)	–	0.92 (0.848–0.953)
All species	48	0.767 (0.475–0.999)	0.93 (0.361–0.999)	0.55 (0–0.998)	0.58 (0.0472–0.998)	0.0021 (0–0.0266)	0.92 (0.636–0.992)

According to species records							
Range	N	AUC	Sensitivity	Specificity	CCR	Und	Ove
T	12	0.974 (0.90–0.997)	0.95 (0.75–0.999)	0.93 (0.870–0.970)	0.93 (0.871–0.970)	0.00020 (0–0.000609)	0.92 (0.738–0.988)
NtNu	22	0.92 (0.732–0.999)	0.86 (0.406–0.999)	0.82 (0.215–0.999)	0.82 (0.231–0.999)	0.0061(0–0.0284)	0.91 (0.667–0.992)
U	14	0.73 (0.659–0.873)	0.66 (0.534–0.854)	0.64 (0–0.758)	0.65 (0.170–0.762)	0.11 (0.00945–0.999)	0.85 (0.740–0.907)
All species	48	0.88 (0.659–0.999)	0.82 (0.406–0.999)	0.79 (0–0.999)	0.79 (0.170–0.999)	0.036 (0–0.999)	0.90 (0.667–0.992)

**Table 3:** Results of the Wilcoxon signed-rank test for matched samples comparing the discrimination (AUC: Area Under the Curve) and classification (Sensitivity, Specificity, CCR or Correct Classification Rate, Und or Under Prediction Rate and Ove or Over Prediction Rate) performance of models based on expert knowledge and models based on species records, for the three groups of species: threatened (T), non-endangered and non-ubiquitous (NtNu), and ubiquitous (U) species separately. St. = Standardized Wilcoxon signed-rank statistic. Sig. = Significance. Negative values of the statistics mean that models based on species records had higher values than those based on expert knowledge

	T		NtNu		U	
	St	Sig	St	Sig	St	Sig
AUC	− 0.471	0.638 (NS)	− 3.806	0.000141	− 3.296	0.000982
Sensitivity	0.405	0.686 (NS)	0.345	0.730 (NS)	3.296	0.000982
Specificity	1.412	0.158 (NS)	− 2.451	0.0142	− 3.180	0.00147
CCR	1.412	0.158 (NS)	− 2.451	0.014	− 3.296	0.001
Und	0.105	0.917 (NS)	− 1.306	0.191 (NS)	–	–
Ove	− 1.647	0.099 (NS)	2.289	0.0022	3.296	0.001

**Table 4:** Results of the Kruskal–Wallis test comparing the discrimination and classification abilities for threatened species (T), non-threatened and non-ubiquitous species (NtNu), and ubiquitous species (U), as well as for all groups together, of the models based on species records and on expert knowledge. AUC: Area Under the ROC Curve. Sens: Sensitivity. Spe: Specificity. CCR: Correct Classification Rate. Und: Underprediction Rate. Ove: Overprediction Rate

	All Groups		T-NtNu		T-U		NtNu-U	
	Est	Sig	Est	Sig	Est	Sig	Est	Sig
AUC-records	29.654	0.001	8.568	0.088 (NS)	28.536	0.0001	19.968	0.0001
AUC-experts	31.447	0.001	11.068	0.026	29.750	0.0001	18.682	0.0001
Sens-records	24.981	0.001	6.386	0.194 (NS)	25.107	0.0001	18.721	0.0001
Sens-experts	16.097	0.001	9.33	0.035	− 7.375	0.129 (NS)	− 16.705	0.0001
Spe-records	27.416	0.001	11.409	0.023	28.357	0.0001	16.948	0.0001
Spe-experts	34.748	0.001	10.947	0.027	31.083	0.0001	20.136	0.0001
CCR-records	27.856	0.001	11.295	0.025	28.536	0.0001	17.240	0.0001
CCR-experts	33.954	0.001	11.011	0.028	31.125	0.0001	20.114	0.0001
Und-records	29.946	0.001	− 8.061	0.101 (NS)	− 27.905	0.0001	− 19.844	0.0001
Und-experts	18.340	0.001	− 11.455	0.010	–	–	–	–
Ove-records	12.521	0.002	0.121	0.981 (NS)	15.810	0.0040	15.688	0.0010
Ove-experts	4.038	0.133(NS)	–	NS	–	NS	–	NS

For the 22 NtNu species, 14 models based on species records showed outstanding discrimination capacity, with another five models showing excellent discrimination (0.8 < AUC < 0.9), while for models based on expert knowledge, only eight models demonstrated outstanding discrimination and six others excellent discrimination. This indicates that models based on species records outperformed those based on expert knowledge (Wilcoxon signed-rank test significance p < 0.001).

Experts did not discriminate between the 14 ubiquitous species (AUC = 0.5), precisely because these species were considered by them to be present throughout Uruguay. However, 10 models based on species records for these species showed acceptable discrimination according to Hosmer & Lemeshow [48] (0.7 < AUC < 0.8) and one showed excellent discrimination (Wilcoxon signed-rank test significance p < 0.001, i.e.); this indicates that models based on species records outperformed those based on expert knowledge).

### Classification capacity of the models

Models, whether based on species records or expert knowledge, had three significantly different levels of classification ability, as measured with the CCR, and performed best for threatened species and worst for ubiquitous species (Table 4, and Figure S1 in Additional file 2). The CCRs of models based on species records were significantly higher than those of models based on expert knowledge for all but T species (Table 3). In fact, for T species, the mean CCR of models based on expert knowledge had a higher value, although not significantly higher, than that of models based on species records, even when there was a low outlier for a species (Tables 2 and 3, and Figure S1 in Additional file 2).

Sensitivity values were generally high for all models (Table 2), but were highest for expert knowledge-based models in all three groups of species, with these differences being significant only for U species (Table 3). However, while this higher sensitivity was obtained at the expense of a higher overprediction rate and a lower specificity for NtNu species, for T species, the higher sensitivity of expert knowledge-based models was obtained with (non-significantly) lower overprediction and higher specificity. Models based on species records had the same sensitivity level for T and NtNu species, with a lower level of sensitivity for U species (Table 4, and Figure S1 in Additional file 2). However, models based on expert inferences showed the same level of sensitivity for T and U species, which is notable given that sensitivity for U species was always 1, with a lower level of sensitivity for the other species (Table 4, and Figure S1 in Additional file 2).

Mean specificity values were always lower than those of corresponding mean sensitivity (Table 2). Additionally, specificity values were significantly higher in record-based models than in expert-based models, except for threatened species (Table 3). Both types of models had three significantly different levels of specificity, being best for threatened species and worst for ubiquitous species (Table 4, and Figure S1 in Additional file 2).

Record-based models underpredicted equally for T and NtNu species, but significantly worse for U species, although always with low underprediction values. However, these models overpredicted less for U species than for the other two groups, which performed equally in this regard. Expert-criteria-based models demonstrated no underprediction rate for ubiquitous species (experts did not predict absence in any location) and underpredicted significantly better for T species than for NtNu species. Models based on expert criteria displayed the same overprediction rate for the three groups of species (Table 4, and Figure S1 in Additional file 2).

### Entropy of the models

The results of the Wilcoxon signed-rank test for matched samples showed that the mean entropy values were significantly lower in models based on expert criteria for threatened species (0.009 vs 0.091, p < 0.01) and for NtNu species (0.035 vs 0.19, p < 0.001). The distribution models of the NtNu species had higher entropy values than those of T species with either the species records or the expert criteria. The models produced using species records for U species had the highest entropy values (0.518); individual entropy values can be seen in the maps of Figure S3 in Additional file 3.

### Combination of the fuzzy sets derived from the models based on records and expert criteria

**Fig. 2:**
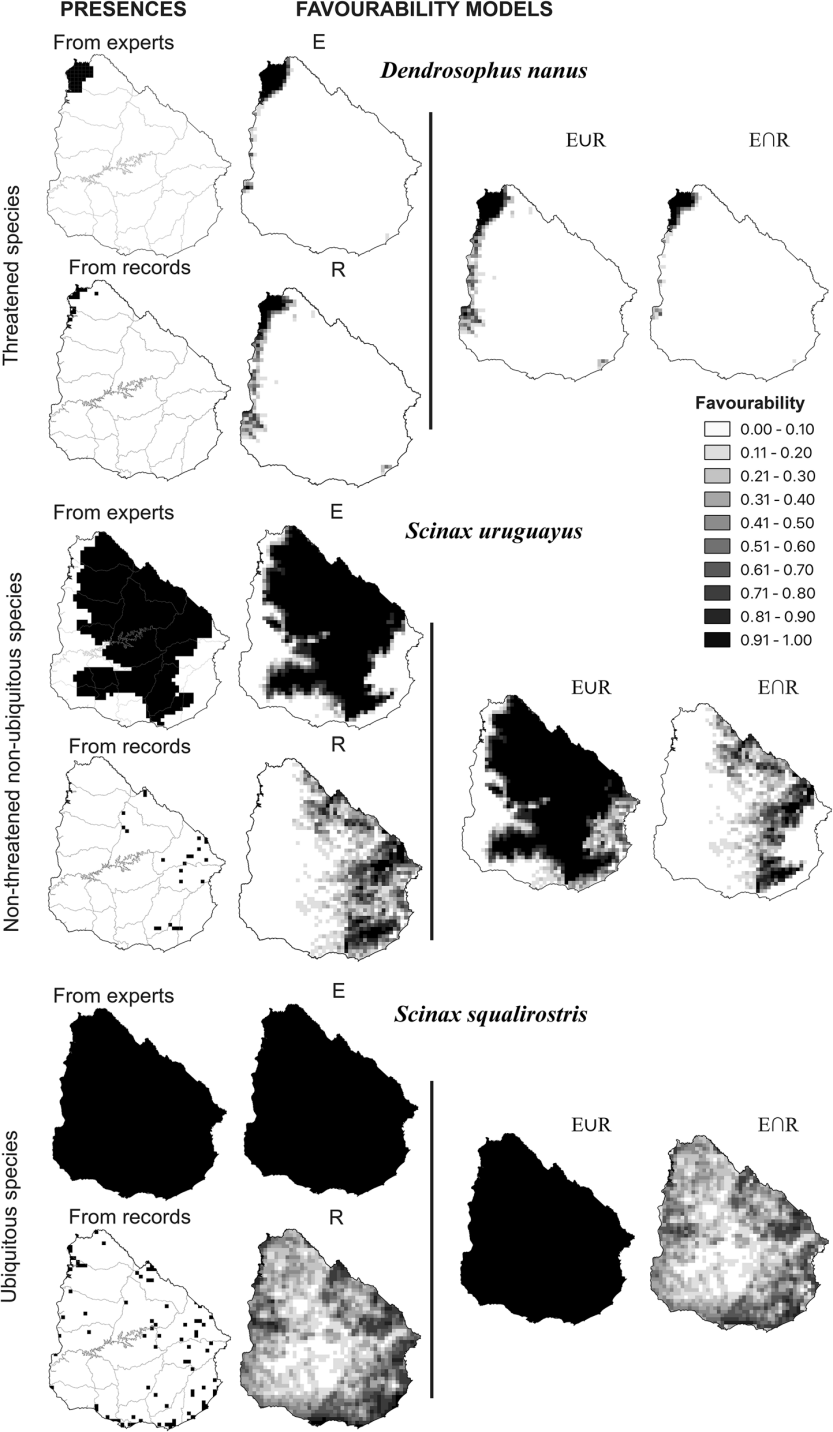
General scheme of the procedure used in three species as examples: *Dendropsophus nanus* (upper part) as a threatened species, *Scinax uruguayus* (middle part) as a non-threatened non-ubiquitous species, and *Scinax squalirostris* (lower part) as a ubiquitous species. From left to right: grid cells with presence according to both sources of information (upper maps: according to expert-criteria; lower maps: according to species records); the favourability models based on expert knowledge (E); the favourability models based on species records (R); their fuzzy union (E ∪ R) and their fuzzy intersection (E ∩ R)

The results of combining the fuzzy set E and R derived from the models based on expert criteria and on recorded distribution, respectively, using their fuzzy union and intersection appear in maps by species of Figure S3 in Additional file 3. Figure 2 shows these results using three species as an example: *Dendrosophus nanus* as a T species, *Scinax uruguayus* as an NtNu species and *Scinax squalirostris* as a U species.

Mean overlap values between fuzzy sets E and R were similar for the three groups of species: 0.34 for T species, 0.37 for NtNu species and 0.42 for U species. For 17% of species, the spatial overlap between both alternative models was higher than 50%. Individual overlap value can be seen in the maps of Figure S3 in Additional file 3.

For threatened species, the mean inclusion of the fuzzy set E in the fuzzy set R was 0.66, whereas the mean inclusion of R in E was 0.40. This suggests that expected distribution according to recorded data was larger than that expected according to expert knowledge. However, for NtNu species, the mean inclusion of E in R was 0.51, whereas the mean inclusion of R into E was 0.67, meaning that, for these species, the distribution expected based on expert knowledge was larger. For U species the mean inclusion of E into R was 0.42, whereas the mean inclusion of R into E was 1, which was expected given that the distribution expected according to expert criteria included all of Uruguay, according to the experts.

## Discussion

When studying the distribution of species, researchers attempt not only to describe, but also to understand and explain the distribution patterns of species. Classical mathematics can be used to this end through the application of statistical methods and precise mathematical models in the study of species distributions [53]. Methods such as regression analysis, cluster analysis and probability theory, among others, can be used to analyse species presence and absence data, assess distribution patterns, and predict future distribution based on environmental and geographic variables. The inclusion of fuzzy logic, which is derived from fuzzy set theory, provides species distribution models with a malleability particularly useful for dealing with the intrinsic uncertainty and vagueness of species distribution data [25, 26]. This is the case because information on the presence or absence of species can often be ambiguous due to the lack of precise data or natural variability in the distribution of the species. [3, 6]. Fuzzy logic allows this kind of ambiguity to be addressed by working with partial truth values (about the species being present at a locality) or degrees of membership in a category (in a species’ range) [5, 25].

Human rationale, when used in the form of expert opinions on distribution data, for example, often yields uncertainty and incomplete information. As a result, it is common to work using degrees of confidence with the information provided. In this manner, although expert knowledge is grounded in empirical observations of species distribution, including both presences and absences recorded in field samples, experts also implicitly use informal fuzzy logic when constructing or completing their mental model of a species’ occupied territory [25, 26]. Fuzzy logic allows formal handling of this uncertainty in human thinking, thus reflecting and making more explicit the lack of certainty in our statements and beliefs. Consequently, fuzzy logic allows work with commensurable categories of the uncertainty and vagueness inherent in the incomplete nature of species distribution data (species records) and in human thinking and beliefs (expert knowledge). Our work suggests that the subjective representation of species distribution by experts may reflect, perhaps unconsciously, perceived relationships between observed occurrences of species and environmental conditions, and that these relationships may be objectively assessed using the formal analysis of fuzzy logic.

Our approach compared models based on both expert knowledge and species records by evaluating their capacity to discriminate and correctly classify species records; this differs from other previous approaches, which have evaluated the correctness of expert-defined ranges on the basis of their similarity with ranges derived from species distribution modelling [34]. The latter approach precludes the possibility that a model based on expert opinion can be better than one produced by mathematical modelling, whereas this is one of the main results in this work.

### General assessments of the models

Although both kinds of models performed well, discriminating and classifying the amphibian occurrences in Uruguay in an acceptable manner, models based on expert knowledge discriminated equally as well as or even better than corresponding models based on species records for threatened species [12, 16–18]. This finding is particularly remarkable because models based on expert knowledge had to discriminate the same set of records that were used to build the models based on species records; it is striking that the former sometimes outperformed the latter. However, experts also used the same overprediction rate for the three types of species, i.e., when experts filled in the gaps within their mental fuzzy model of species distribution [34, 36], they did so equally for all types of species. Nonetheless, this kind of generalisation is useful for discriminating the distribution range of threatened species, while it yields uninformative models for widespread species. In other words, expert judgment, although consistent, is more useful when dealing with threatened, typically specialist species and less useful when dealing with more generalist species. Conversely, objective species distribution models were more valuable when dealing with generalist species than when approaching specialist species that may be well understood by experts.

### Drivers of the geographical range of amphibians

These models, built on the basis of two different sources of information, incorporated factors that have been identified in the literature as significant drivers of amphibian distributions in various regions [23, 54, 55], including Uruguay [17, 56–58]. Furthermore, the factors better represented in the models from both sources of information were those that exerted influence on species distribution at large spatial scales (climate, topography, land uses and spatial location), ranging from landscape (10–200 km) to regional and national scales (from 200 km to greater than 2000 km) [38]. However, models based on the two sources of information differed in the number of factors and variables invoked to explain the distribution of amphibian species, as the models based on the knowledge of the experts were more complex. This was unexpected, given that the experts produced distribution ranges apparently simpler and more homogeneous than the distribution pattern of species records. However, despite the higher complexity of expert-based models, they resulted in models with lower entropy. Consequently, these models proposed more structured and organized patterns of favourable territories for each species, in comparison to models based on recorded distributions.

It is worth noting that human activity was identified as an explanatory factor in many of the models based on both information sources. When the human variables in the models indicated a higher favourably with proximity to roads and urban centres, it mostly revealed biases in the sampled records. This was the case because researchers tend to sample more frequently in accessible and well-connected territories [12, 13, 59, 60]. Expert-based models were less affected by this kind of sampling biases [59], which may be related to the generalization capacity of the experts allowing them to dodge the effects of the sampling bias. Consequently, expert knowledge may be most valuable in countries with high biodiversity, which are often undersampled, especially for taxa that are generally underestimated, such as amphibians [61].

### Comparing the extent of models based on expert criteria and field records

Fuzzy overlap between the two kinds of models was notably low, in the range 0.34–0.42. This suggests that the areas considered as favourable for the species according to the two types of information differed substantially. The inclusion of one type of model int the other, i.e., the degree to which the model based on expert knowledge was a subset of the model based on species records and vice versa, differed according to the types of species analysed. For threatened species, the models based on expert knowledge were mostly a subset of the corresponding models based on species records, while the opposite occurred for non-threatened species.

Some authors have highlighted the tendency of experts’ fuzzy thinking to incorporate absences areas as favourable territories [34], rather than excluding presence records outside the predicted favourable areas. However, our results indicate that both types of models, whether based on expert thinking or on species records, did this, and that experts were less prone to this kind of overprediction for threatened species, as specificity values were higher for expert-derived models of threatened species. The fuzzy set of favourable territories derived from expert-based models encompasses the favourable territories indicated by field records only for species with records extended over wide territories (NtNu and U). Although some authors have suggested that experts tend to think of the environmental territories occupied by these species as polygons that should include all known records [34, 36, 62–64], and that the set of favourability territories derived from records is more fitted to the specific environmental conditions highlighted by punctual records [34, 36], our results suggest that this rationale is only valid for the two types of non-threatened species. For threatened species (see species in Table S1 in Additional file 1), expert models predicted more restrictive fuzzy sets of favourable territories than those predicted by the records themselves. This could be attributed to the fact that threatened species generally exhibit a narrower distribution and, thus, occupy narrower environmental ranges [18, 19, 32, 33], as well as the fact that greater research effort and interest is typically dedicated to acquiring information on threatened species, which are usually better known by experts.

### Bridging distribution gaps with expert knowledge and distribution modelling

For both kinds of models, the patterns of favourable zones completed the absence gaps between zones with presences [65]. Thus, the favourable zones were indicative of a distribution that is less dispersed and discontinuous than that suggested by the known records, and which fills gaps in the currently known distribution. This lack of knowledge concerning distributions could affect the conservation of these fauna groups. Therefore, although knowledge of species distributions has increased in recent decades [66], sampling efforts need to be strengthened in relation to large groups of fauna to obtain a more complete range of sampling-confirmed records and to be able to better define the territories occupied by species [65–67]. Even so, our results suggest that expert knowledge is particularly useful for assessing the distribution of threatened species, which are of greatest conservation concern, and should be more appreciated in areas with fewer funds devoted to sampling efforts.

On the other hand, according to expert criteria about thirty percent of Uruguay amphibian species occupy territories throughout all of the country [17]. However, favourable zones identified by models based on sampling records made it clear that, for these widely distributed amphibian species, there are zones of low or intermediate environmental favourability in which these species are probably absent or present in lower abundances [9, 68]. Although experts overestimate the distributions of species in the same way irrespective of species status as threatened or not, for widespread species, this overprediction leads to uninformative prediction (ubiquity of the distribution). In contrast, models based on observed records had a significantly lower overprediction rate for ubiquitous species than for the other groups for species, helping them to provide better information on the nuances of the distribution patterns of widespread species [5, 9, 55, 69].

### Combination of models based on experts and on records

The use of fuzzy methods, such as favourability functions, to analyse species distributions provides fuzzy degree values to interpret the occurrence of species, yielding more dynamic results than those provided by classical modelling techniques [5, 6]. One new possibility is to combine models based on records and on expert knowledge. Some researchers have integrated recorded occurrences and expert data into the same modelling approach [36], or compared models resulting from both approaches [9, 34]. Fuzzy logic, rather, allows the combination of the models based on the two separate information sources [5, 44]. The application of fuzzy intersection defined areas that were consider favourable in the two types of models. This generally resulted in distribution limits mainly established by expert knowledge for threatened species, whereas they were more similar to the models based on recorded data for the rest of species (Fig. 2). Thus, this method of achieving a consensus between models based on the two kinds of information is able to give a higher weight to the best model for each species.

## Conclusions

In line with the findings of other authors [9, 11, 34, 36], this study emphasizes the crucial role of incorporating expert judgment in distribution models. In the case of threatened species, the expert opinion on these species allowed for a more nuanced and accurate representation of the habitat requirements of the species and the factors that influence their distribution [57]. The use of fuzzy logic provides more dynamic tools that can be used to study the uncertain distribution of biodiversity, allowing extraction of the best information from observation records and expert knowledge. Our analysis demonstrated quantitatively that the fuzzy set of favourable areas predicted from expert knowledge was as accurate as that predicted by field records themselves, and even better in the case of threatened species. In addition, the models based on expert criteria encompassed a greater explanatory complexity to define the requirements of these species. For generalist species, models based on observed data were more accurate.

This approach provides relevant information when planning territories to be protected, and highlights territories in which sampling efforts should be improved to assess the state of conservation of the biodiversity of these areas. We propose that the fuzzy union between the models based on record data and on maps provided by experts can be used to design a national sampling strategy in Uruguay. This would validate the applicability of this new methodology for other fauna groups and regions.

### Supplementary Information

**Additional file 1: Table S1** shows the list of amphibian species analysed in Uruguay, including the IUCN threat category.

**Additional file 2: Figure S1** shows the results of the Kruskal–Wallis test comparing the discrimination and classification abilities for threatened (T), non-threatened and non-ubiquitous (NtNu) and ubiquitous species (U) of the models based on expert knowledge and species records, respectively. **Figure S2** compares the average number of factors, average number of variables and entropy values of the models based on expert knowledge and species records.

**Additional file 3: Figure S3** represents cartographically the occurrences and favourability model values for all amphibian species analyzed in Uruguay according to expert criteria and to species records.

## Data Availability

The dataset supporting the conclusions of this article is available at 10.5061/dryad.0k6djhb6g.
