# Correction: Using fuzzy logic to compare species distribution models developed on the basis of expert knowledge and sampling records

**DOI:** 10.1186/s12983-024-00539-x

**Published:** 2024-06-25

**Authors:** David Romero, Raúl Maneyro, José Carlos Guerrero, Raimundo Real

**Affiliations:** 1 Biogeography, Diversity, and Conservation Research Team, Department of Animal Biology, Faculty of Sciences, Universidad de Málaga, Málaga, Spain; 2 Laboratory of Systematics and Natural History of Vertebrates, Faculty of Sciences, Universidad de La República, Montevideo, Uruguay; 3 Laboratory for Sustainable Development and Environmental Management, Faculty of Sciences, Universidad de La República, Montevideo, Uruguay

**Correction** **:** **Front Zool 20, 38 (2023)**

**https://doi.org/10.1186/s12983-023-00515-x**

Following publication of the original article [1], the authors reported that the link to the data repository is missing in section Availability of data and materials.

The original content of Availability of data and materials was:

The dataset supporting the conclusions of this article will be made available through a data repository.

The correction content should read:

The dataset supporting the conclusions of this article is available at 10.5061/dryad.0k6djhb6g.

The original article [1] has been updated.
