# Supplementary material for: Using fuzzy logic to compare species distribution models developed on the basis of expert knowledge and sampling records: Expert knowledge versus sampling in species distribution modelling
Source: Front Zool. 2023 Dec 7;20:38. doi: 10.1186/s12983-023-00515-x (PMC10702020; doi:10.1186/s12983-023-00515-x)
Supplement: Supplementary file 1 — Additional file 1: Table S1 shows the list of amphibian species analysed in Uruguay, including the IUCN threat category. [file 12983_2023_515_MOESM1_ESM.docx]

**Frontiers in Zoology**

SUPPLEMENTARY INFORMATION

**Title: Using fuzzy logic to compare species distribution models developed on the basis of expert knowledge and sampling records**

*Expert knowledge versus sampling in species distribution modelling*

**Authors:** Romero David ^1*^, Maneyro Raúl ^2^, Guerrero José Carlos ^3^ & Real Raimundo ^1^

**Affilations:** ^1^ Biogeography, Diversity, and Conservation Research Team, Department of Animal Biology, Faculty of Sciences, Universidad de Málaga, Málaga, Spain; ^2^ Laboratory of Systematics and Natural History of Vertebrates, Faculty of Sciences, Universidad de la República, Montevideo, Uruguay; ^3^ Laboratory for Sustainable Development and Environmental Management, Faculty of Sciences, Universidad de la República, Montevideo, Uruguay.

*Corresponding author: davidrp@uma.es

**Additional file 1**

**Table S1.** List of amphibian species analyzed in Uruguay. For each species is shown the current threat category according to the IUCN criteria, and the number of 10 x 10 km squares with presences according to both sources of distribution analysed: based on expert knowledge (N-Pres Experts) and on sampling records (N-Pres Records) (1887 total grid cells conform the Uruguay country). We mark in parentheses the name prior to the latest taxonomic updates. *Threat codes; CR: Critical endangered, EN: Endangered, VU: Vulnerable, NT: Near threatened, LC: Minor concern and DD: Deficient data. **Species status; T: threatened species, Nt-Nu: non-threatened non-ubiquitous species and U: ubiquitous species.

| Species | *Threat | N-Pres Experts | N-Pres Records | **Status |
| --- | --- | --- | --- | --- |
| *Melanophryniscus langonei* | CR | 65 | 2 | T |
| *Melanophryniscus montevidensis* | CR | 74 | 38 | T |
| *Melanophryniscus devincenzii* | EN | 33 | 7 | T |
| *Melanophryniscus pachyrhynus* | EN | 22 | 5 | T |
| *Ceratophrys ornata* | VU | 31 | 9 | T |
| *Dendropsophus minutus* | EN | 88 | 17 | T |
| *Dendropsophus nanus* | EN | 49 | 14 | T |
| *Lysapsus limellum* | EN | 62 | 4 | T |
| *Scinax nasicus* | EN | 137 | 19 | T |
| *Scinax aromothyella*  (*Ololygon aromothyella*) | EN | 248 | 4 | T |
| *Physalaemus fernandezae* | EN | 105 | 4 | T |
| *Leptodactylus furnarius* | CR | 49 | 3 | T |
|  |  |  |  |  |
| *Chthonerpeton indistinctum* | LC | 316 | 15 | Nt-Nu |
| *Melanophryniscus atroluteus* | LC | 1333 | 36 | Nt-Nu |
| *Melanophryniscus sanmartini* | NT | 434 | 10 | Nt-Nu |
| *Rhinella achavali* | NT | 742 | 13 | Nt-Nu |
| *Rhinella arenarum* | LC | 504 | 45 | Nt-Nu |
| *Rhinella dorbignyi* | LC | 910 | 52 | Nt-Nu |
| *Rhinella diptycha*  (*Rhinella schneideri*) | LC | 176 | 30 | Nt-Nu |
| *Odontophrynus maisuma* | NT | 60 | 6 | Nt-Nu |
| *Scinax berthae*  (*Ololygon berthae*) | LC | 775 | 14 | Nt-Nu |
| *Scinax fuscovarius* | LC | 309 | 24 | Nt-Nu |
| *Scinax uruguayus* | LC | 1165 | 23 | Nt-Nu |
| *Phyllomedusa iheringii* | LC | 812 | 28 | Nt-Nu |
| *Physalaemus biligonigerus* | LC | 1289 | 101 | Nt-Nu |
| *Physalaemus gracilis* | LC | 1001 | 68 | Nt-Nu |
| *Physalaemus henselii* | LC | 1532 | 70 | Nt-Nu |
| *Physalaemus riograndensis* | LC | 1611 | 51 | Nt-Nu |
| *Pleurodema bibroni* | NT | 981 | 41 | Nt-Nu |
| *Leptodactylus macrosternum*  (*Leptodactylus chaquensis*) | LC | 135 | 26 | Nt-Nu |
| *Nyctimantis siemersi*  (*Argenteohyla siemersi*) | DD | 24 | 2 | Nt-Nu |
| *Boana albopunctata*  (*Hypsiboas albopunctatus*) | DD | 9 | 1 | Nt-Nu |
| *Physalaemus cuvieri* | DD | 8 | 1 | Nt-Nu |
| *Leptodactylus podicipinus* | DD | 25 | 4 | Nt-Nu |
|  |  |  |  |  |
| *Rhinella fernandezae* | LC | 1883 | 89 | U |
| *Odontophrynus americanus* | LC | 1883 | 172 | U |
| *Limnomedusa macroglossa* | LC | 1883 | 172 | U |
| *Dendropsophus sanborni* | LC | 1883 | 101 | U |
| *Boana pulchella*  (*Hypsiboas pulchellus*) | LC | 1883 | 287 | U |
| *Pseudis minuta* | LC | 1883 | 212 | U |
| *Scinax granulatus* | LC | 1883 | 139 | U |
| *Scinax squalirostris* | LC | 1883 | 94 | U |
| *Pseudopaludicola falcipes* | LC | 1883 | 203 | U |
| *Leptodactylus gracilis* | LC | 1883 | 93 | U |
| *Leptodactylus latinasus* | LC | 1883 | 211 | U |
| *Leptodactylus luctator*  (*Leptodactylus latrans*) | LC | 1883 | 194 | U |
| *Leptodactylus mystacinus* | LC | 1883 | 118 | U |
| *Elachistocleis bicolor* | LC | 1883 | 94 | U |
