# Supplementary material for: Using fuzzy logic to compare species distribution models developed on the basis of expert knowledge and sampling records: Expert knowledge versus sampling in species distribution modelling
Source: Front Zool. 2023 Dec 7;20:38. doi: 10.1186/s12983-023-00515-x (PMC10702020; doi:10.1186/s12983-023-00515-x)
Supplement: Supplementary file 2 — Additional file 2: Figure S1 shows the results of the Kruskal–Wallis test comparing the discrimination and classification abilities for threatened (T), non-threatened and non-ubiquitous (NtNu) and ubiquitous species (U) of the models based on expert knowledge and species records, respectively. Figure S2 compares the average number of factors, average number of variables and entropy values of the models based on expert knowledge and species records. [file 12983_2023_515_MOESM2_ESM.docx]

**Frontiers in Zoology**

SUPPLEMENTARY INFORMATION

**Title: Using fuzzy logic to compare species distribution models developed on the basis of expert knowledge and sampling records**

*Expert knowledge versus sampling in species distribution modelling*

**Authors:** Romero David ^1*^, Maneyro Raúl ^2^, Guerrero José Carlos ^3^ & Real Raimundo ^1^

**Affilations:** ^1^ Biogeography, Diversity, and Conservation Research Team, Department of Animal Biology, Faculty of Sciences, Universidad de Málaga, Málaga, Spain; ^2^ Laboratory of Systematics and Natural History of Vertebrates, Faculty of Sciences, Universidad de la República, Montevideo, Uruguay; ^3^ Laboratory for Sustainable Development and Environmental Management, Faculty of Sciences, Universidad de la República, Montevideo, Uruguay.

*Corresponding author: davidrp@uma.es

**Affilations:** ^1^ Biogeography, Diversity, and Conservation Research Team, Department of Animal Biology, Faculty of Sciences, Universidad de Málaga, Málaga, Spain; ^2^ Laboratory of Systematics and Natural History of Vertebrates, Faculty of Sciences, Universidad de la República, Montevideo, Uruguay; ^3^ Laboratory for Sustainable Development and Environmental Management, Faculty of Sciences, Universidad de la República, Montevideo, Uruguay.

*Corresponding author: davidrp@uma.es

**Additional file 2**


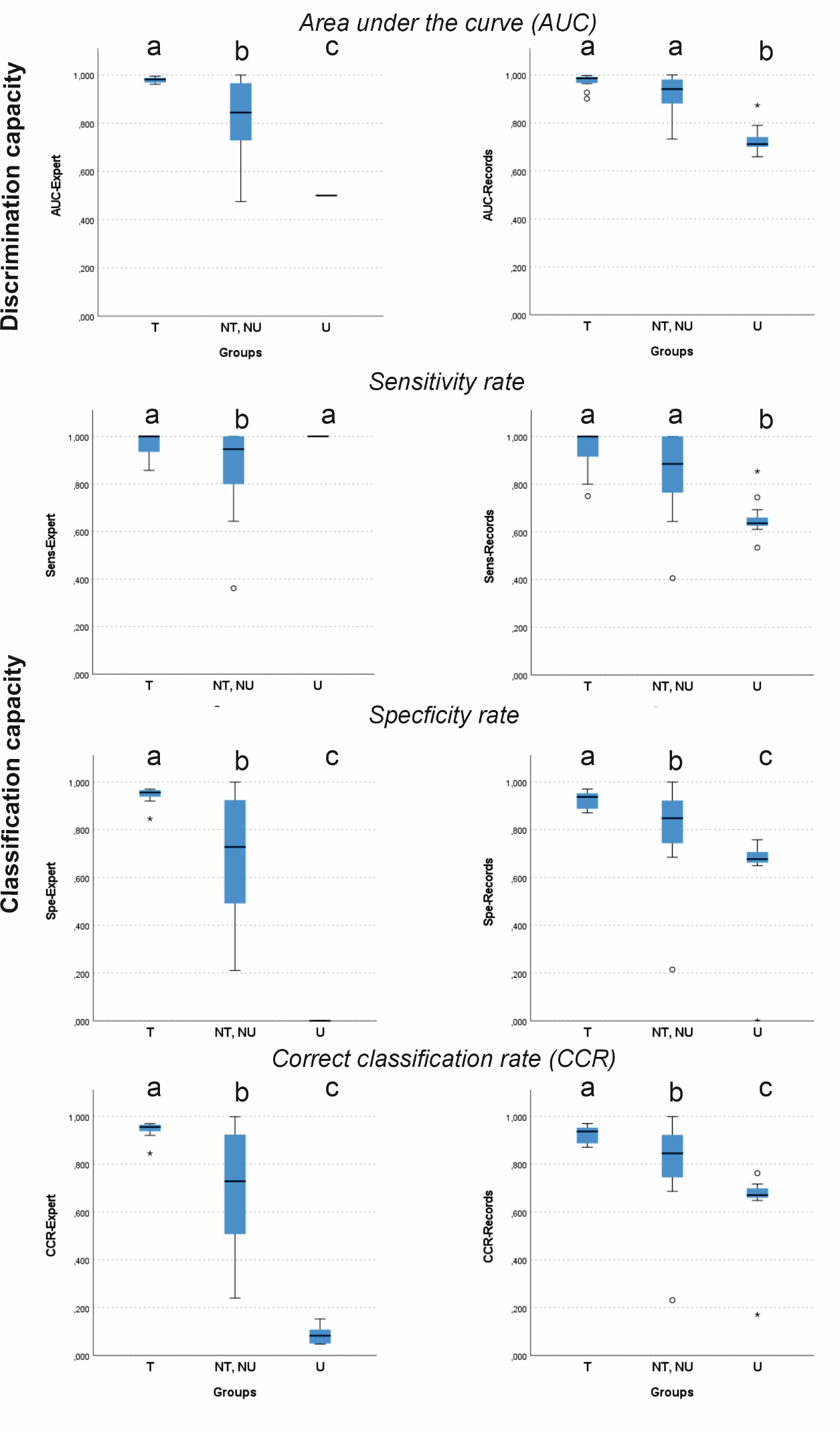


**
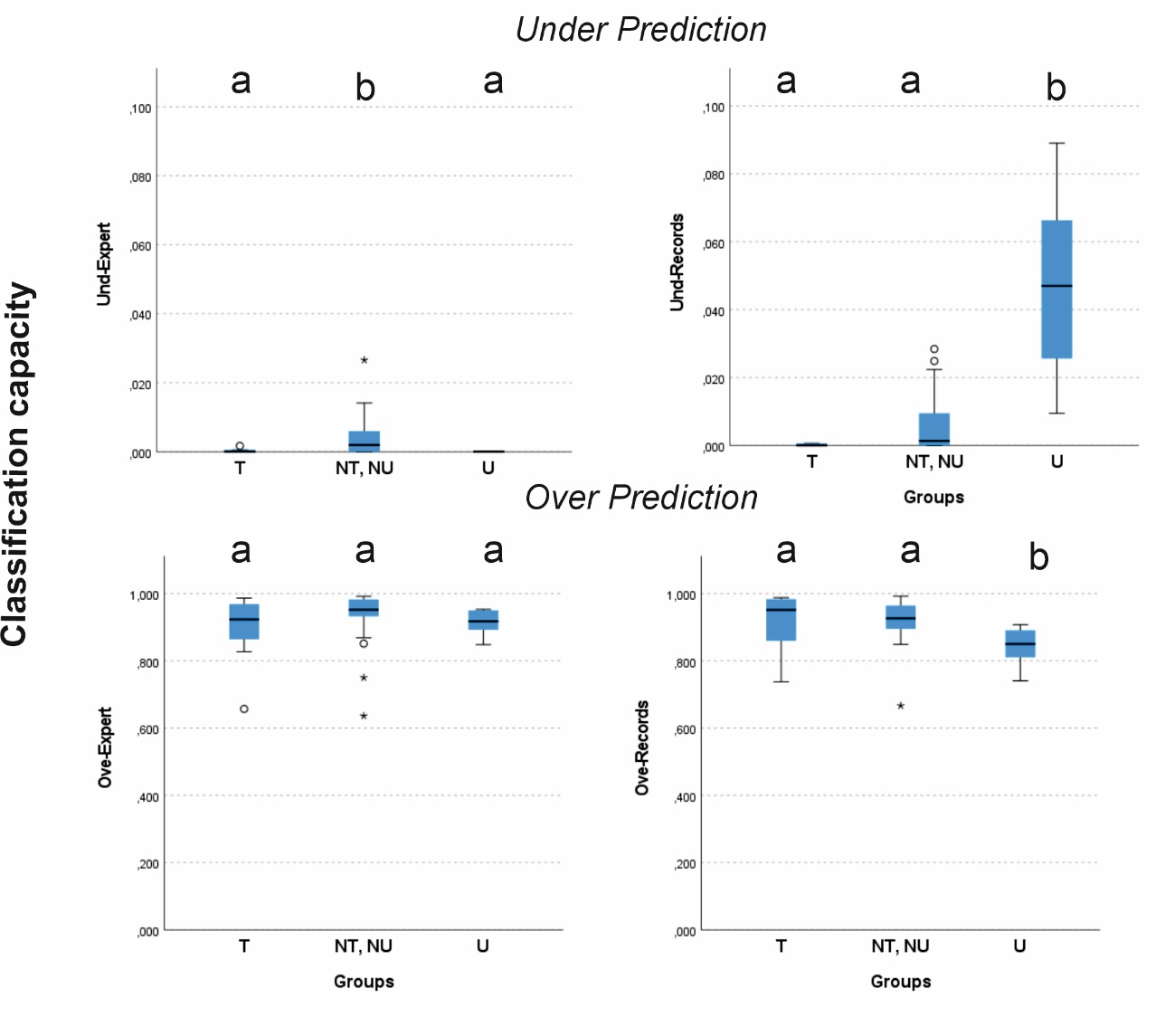
**

**Figure S1.** Results of the Kruskal-Wallis test comparing the discrimination (AUC) and classification abilities for threatened (T), non-endangered and non-ubiquitous (Nt-Nu), and ubiquitous species (U), of the models based on expert knowledge and species records. Species groups without significant differences in performance according to the Kruskal-Wallis test share the same superscript letter.


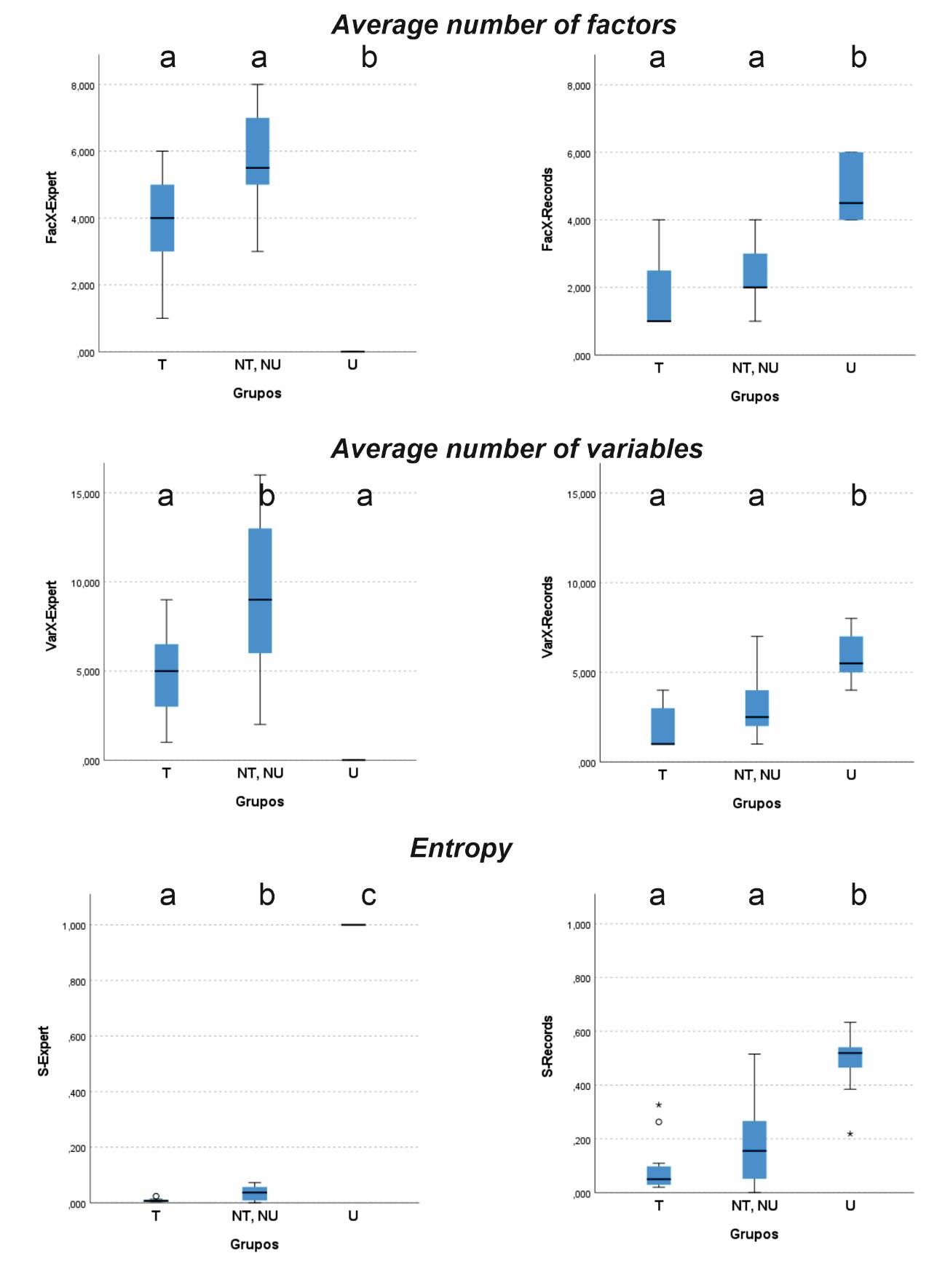


**Figure S2.** Results of the Kruskal-Wallis test comparing the average number of factors, average number of variables and Entropy values for threatened (T), non-endangered and non-ubiquitous (Nt-Nu) and ubiquitous (U) species of the models based on expert knowledge and species records. Species groups without significant differences in performance according to the Kruskal-Wallis test share the same superscript letter.
