# Supplementary material for: Using fuzzy logic to compare species distribution models developed on the basis of expert knowledge and sampling records: Expert knowledge versus sampling in species distribution modelling
Source: Front Zool. 2023 Dec 7;20:38. doi: 10.1186/s12983-023-00515-x (PMC10702020; doi:10.1186/s12983-023-00515-x)
Supplement: Supplementary file 3 — Additional file 3: Figure S3 represents cartographically the occurrences and favourability model values for all amphibian species analyzed in Uruguay according to expert criteria and to species records. [file 12983_2023_515_MOESM3_ESM.docx]

**Frontiers in Zoology**

SUPPLEMENTARY INFORMATION

**Title: Using fuzzy logic to compare species distribution models developed on the basis of expert knowledge and sampling records**

*Expert knowledge versus sampling in species distribution modelling*

**Authors:** Romero David ^1*^, Maneyro Raúl ^2^, Guerrero José Carlos ^3^ & Real Raimundo ^1^

**Affilations:** ^1^ Biogeography, Diversity, and Conservation Research Team, Department of Animal Biology, Faculty of Sciences, Universidad de Málaga, Málaga, Spain; ^2^ Laboratory of Systematics and Natural History of Vertebrates, Faculty of Sciences, Universidad de la República, Montevideo, Uruguay; ^3^ Laboratory for Sustainable Development and Environmental Management, Faculty of Sciences, Universidad de la República, Montevideo, Uruguay.

*Corresponding author: davidrp@uma.es

**Additional file 3**


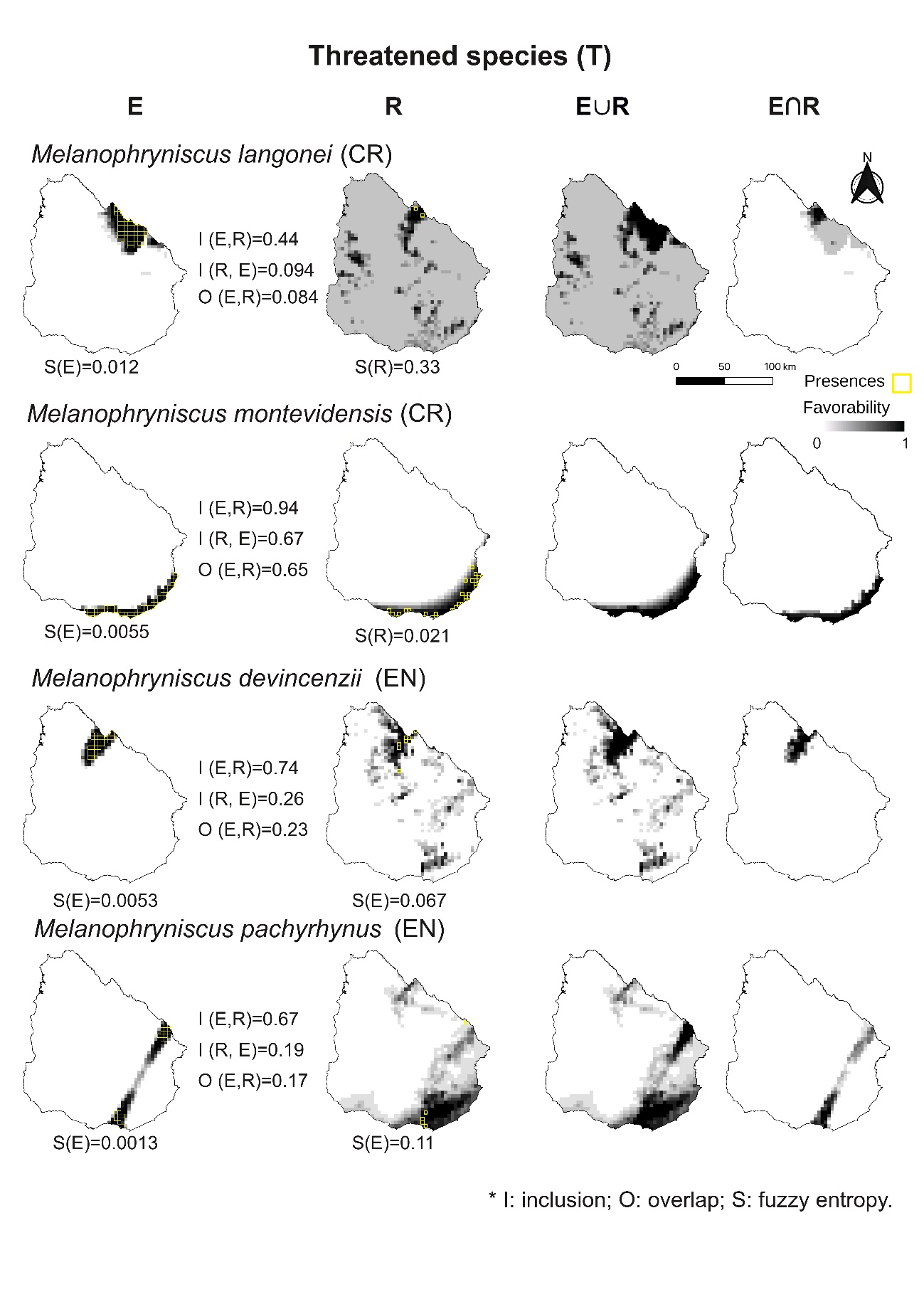

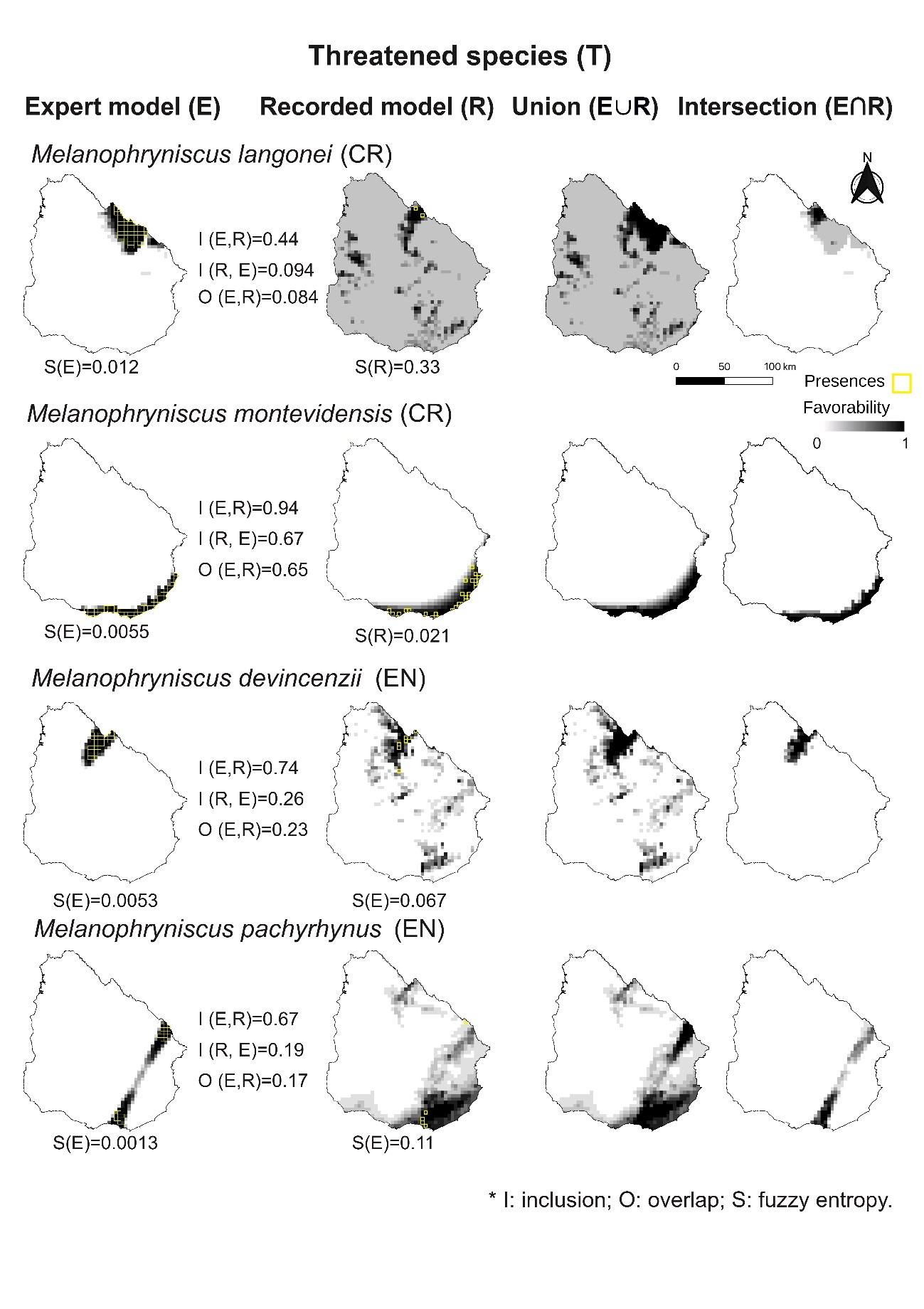
1


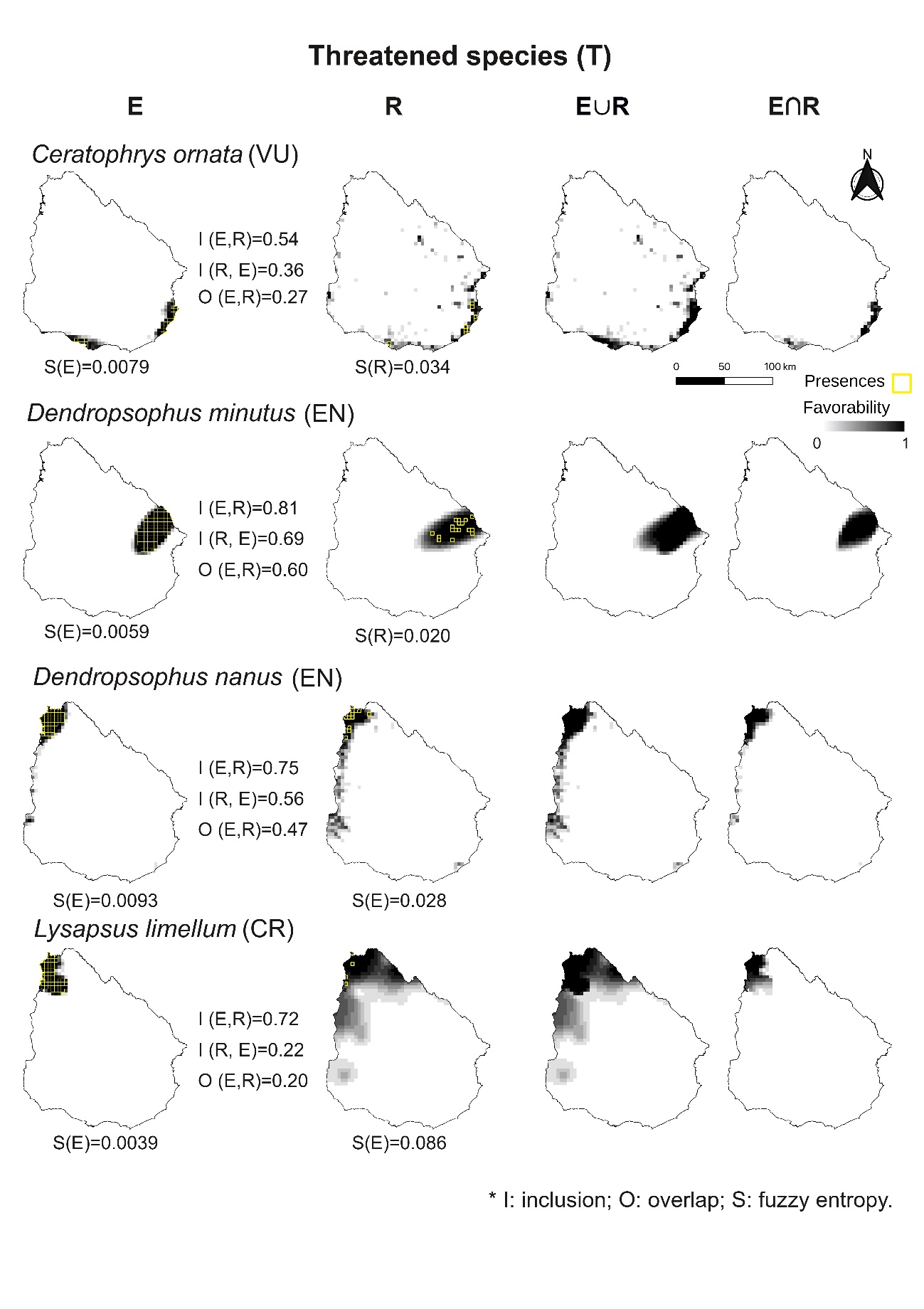

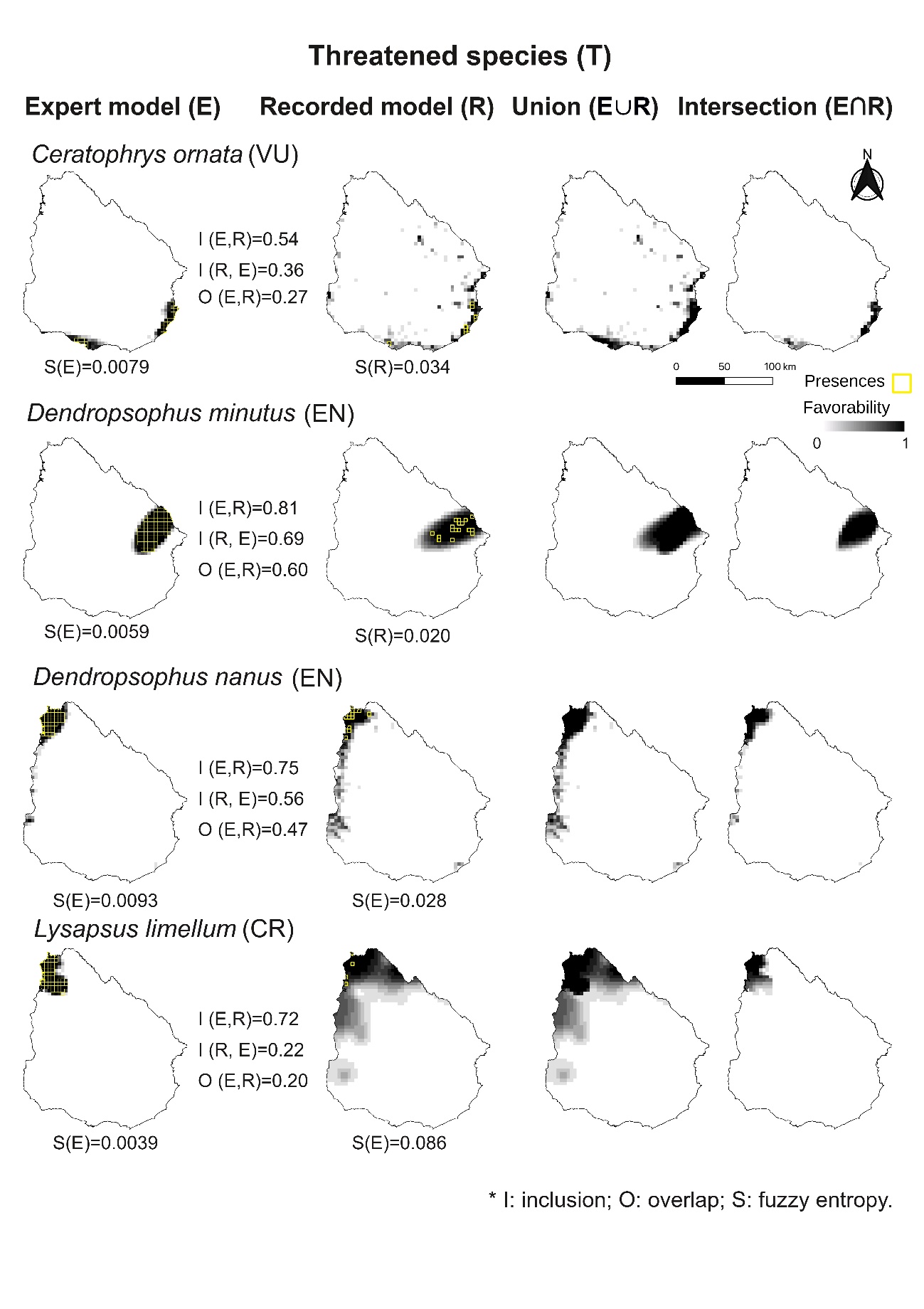
2


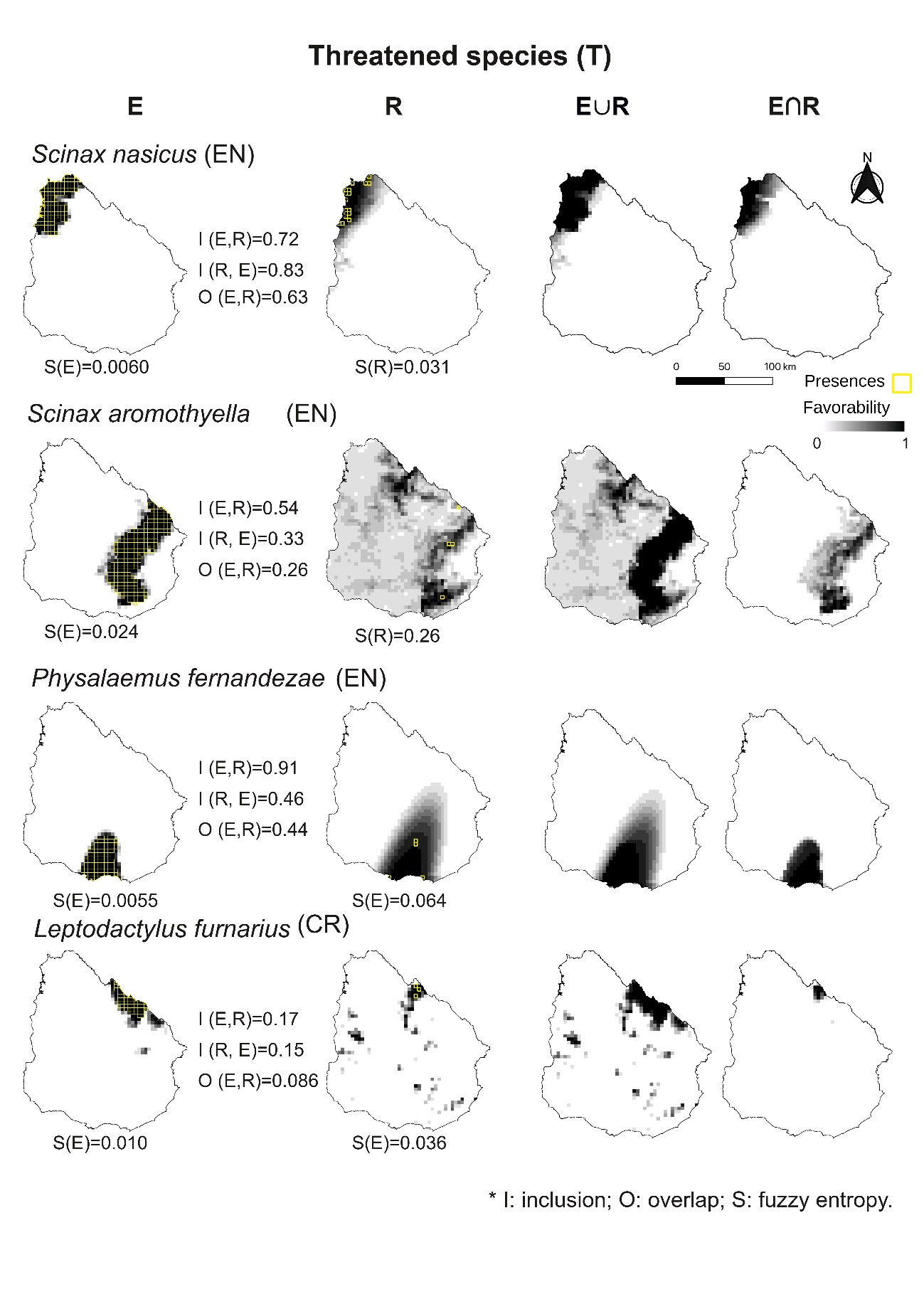


3


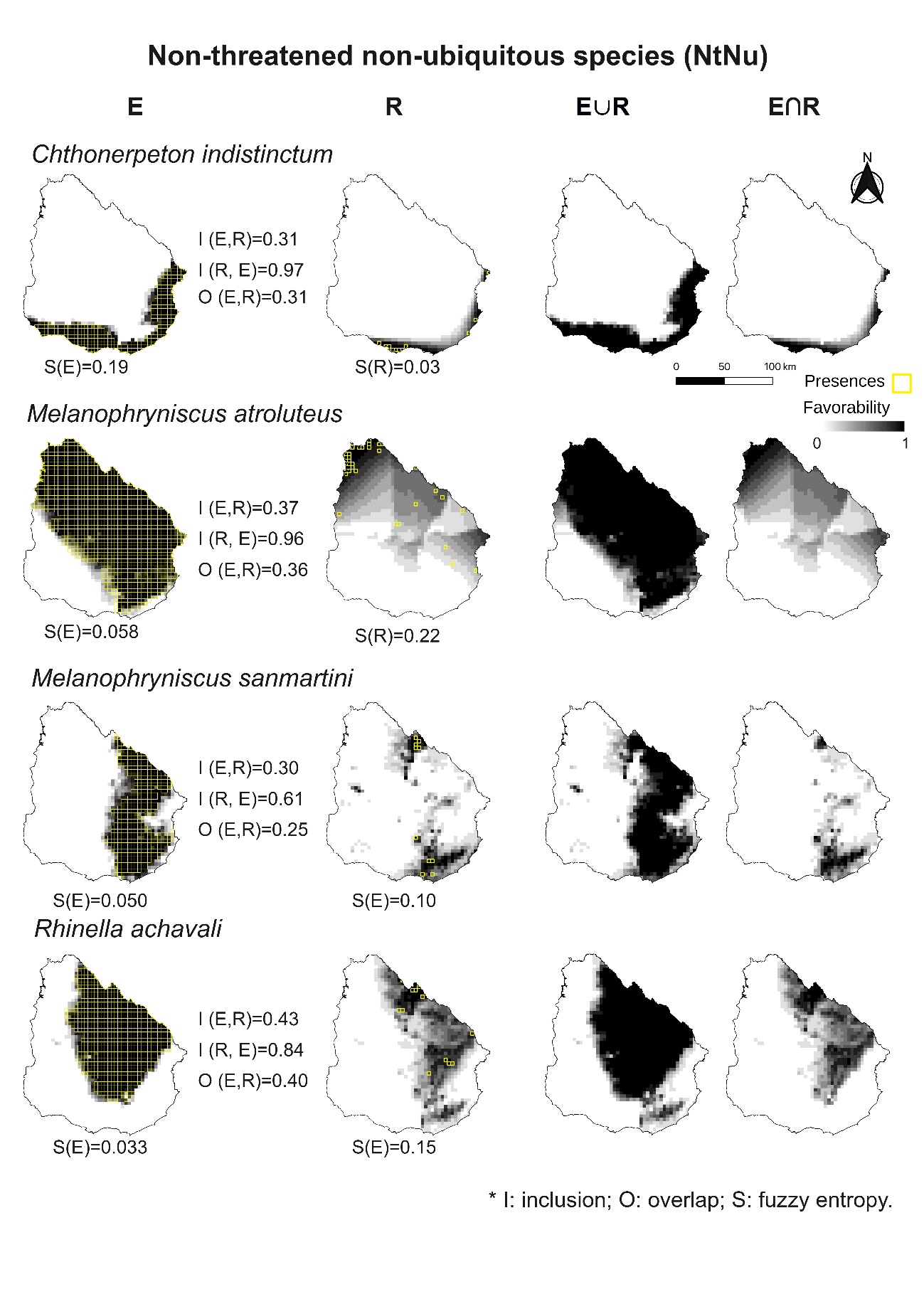


4


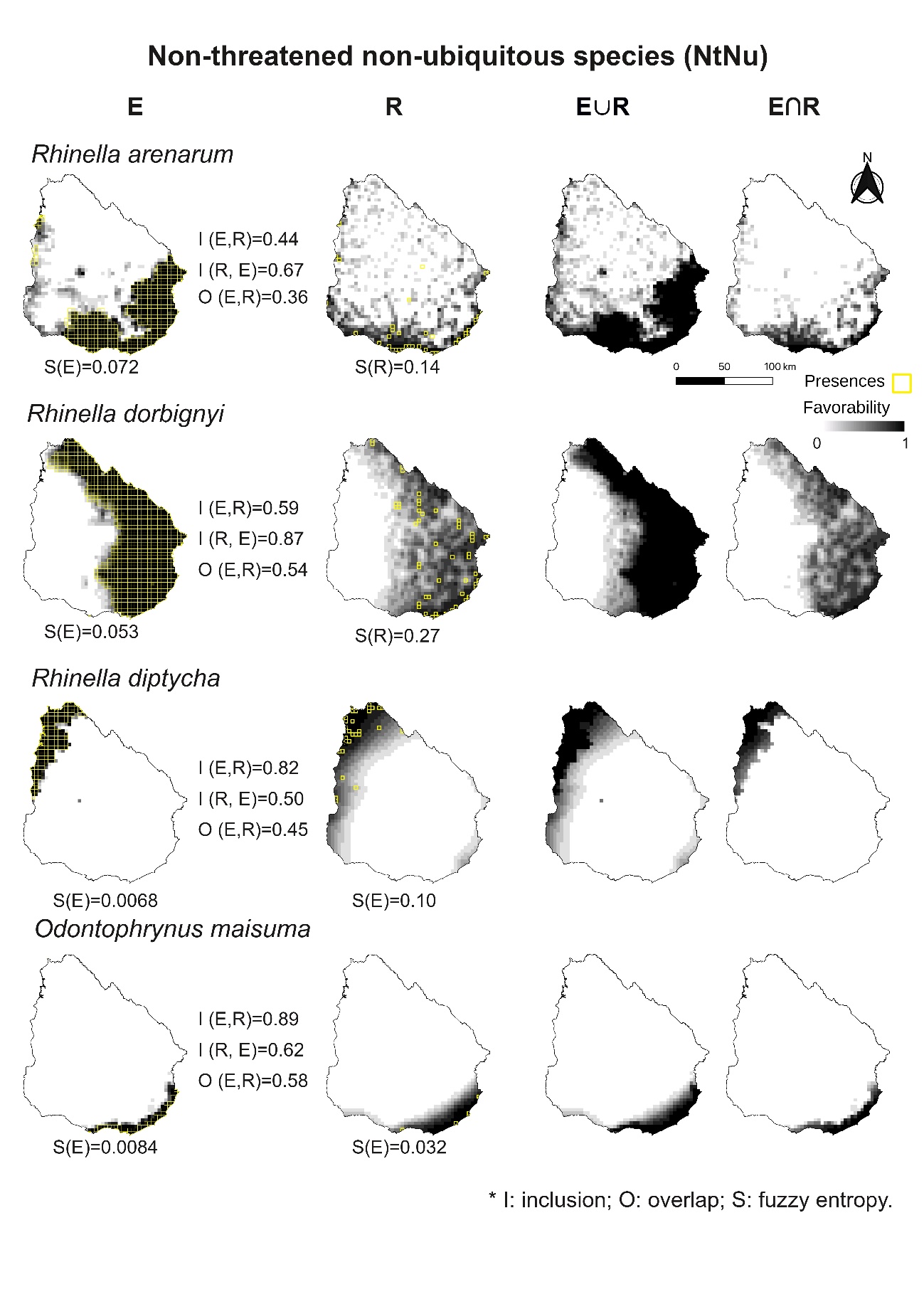


5


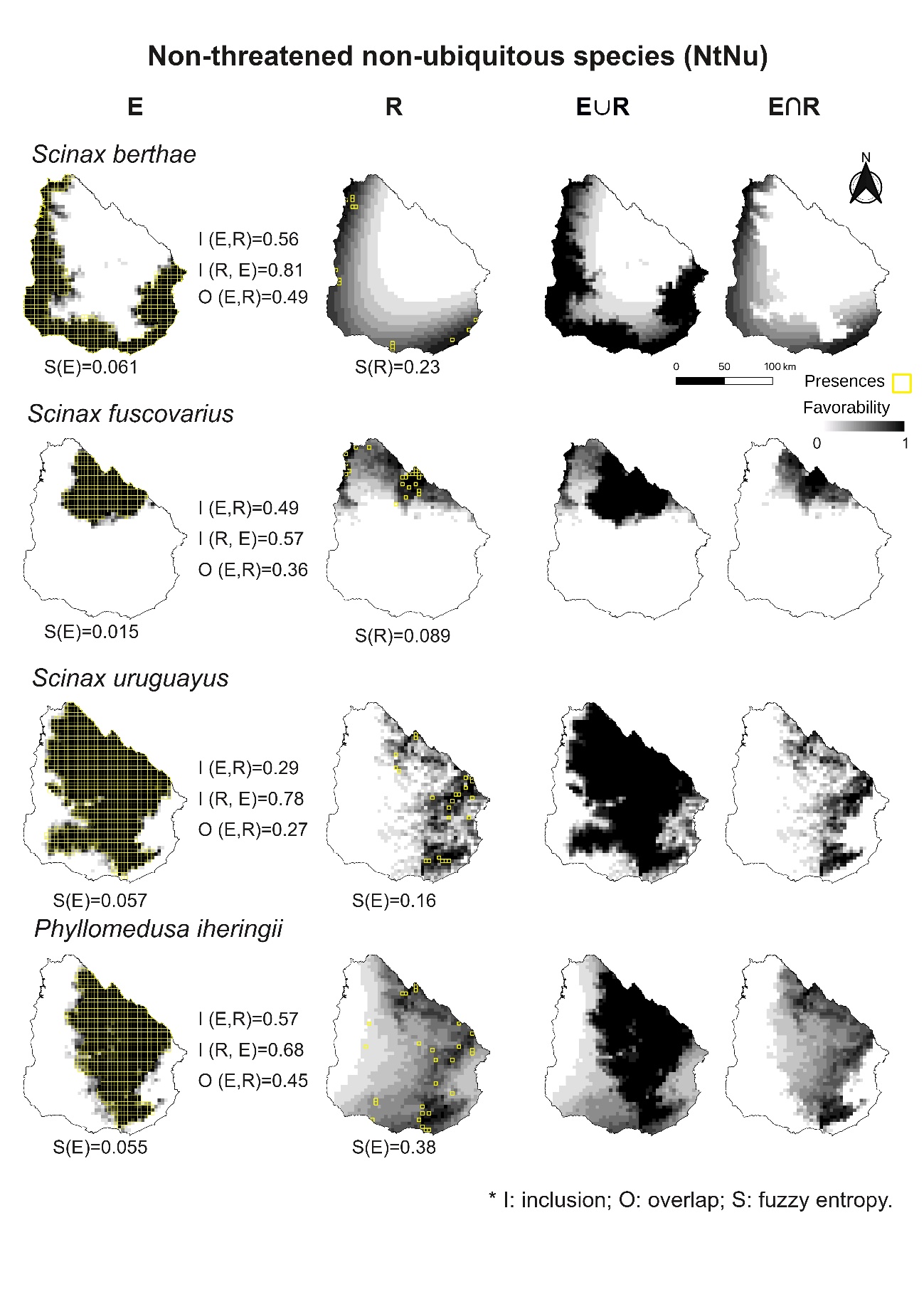
6


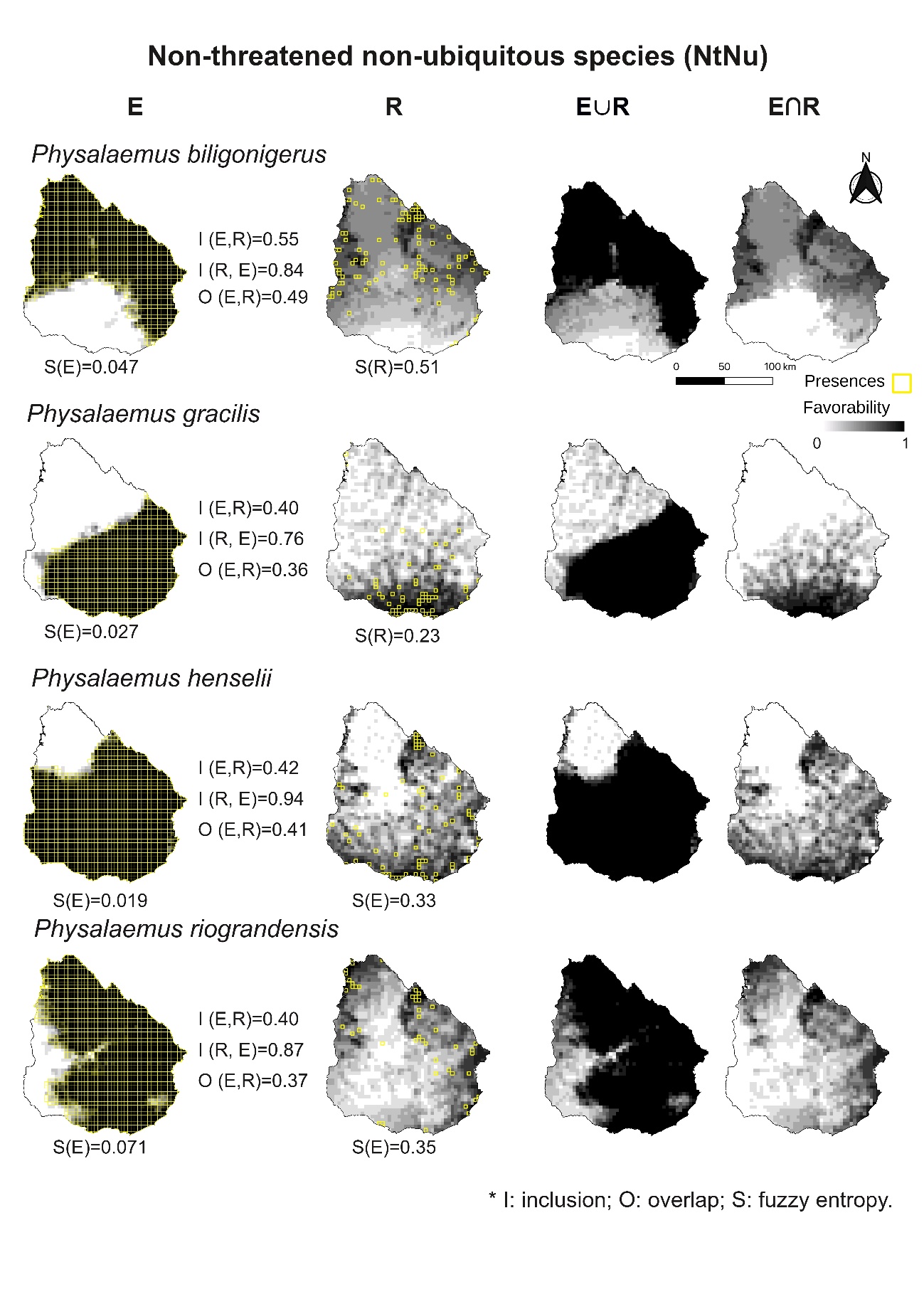


7


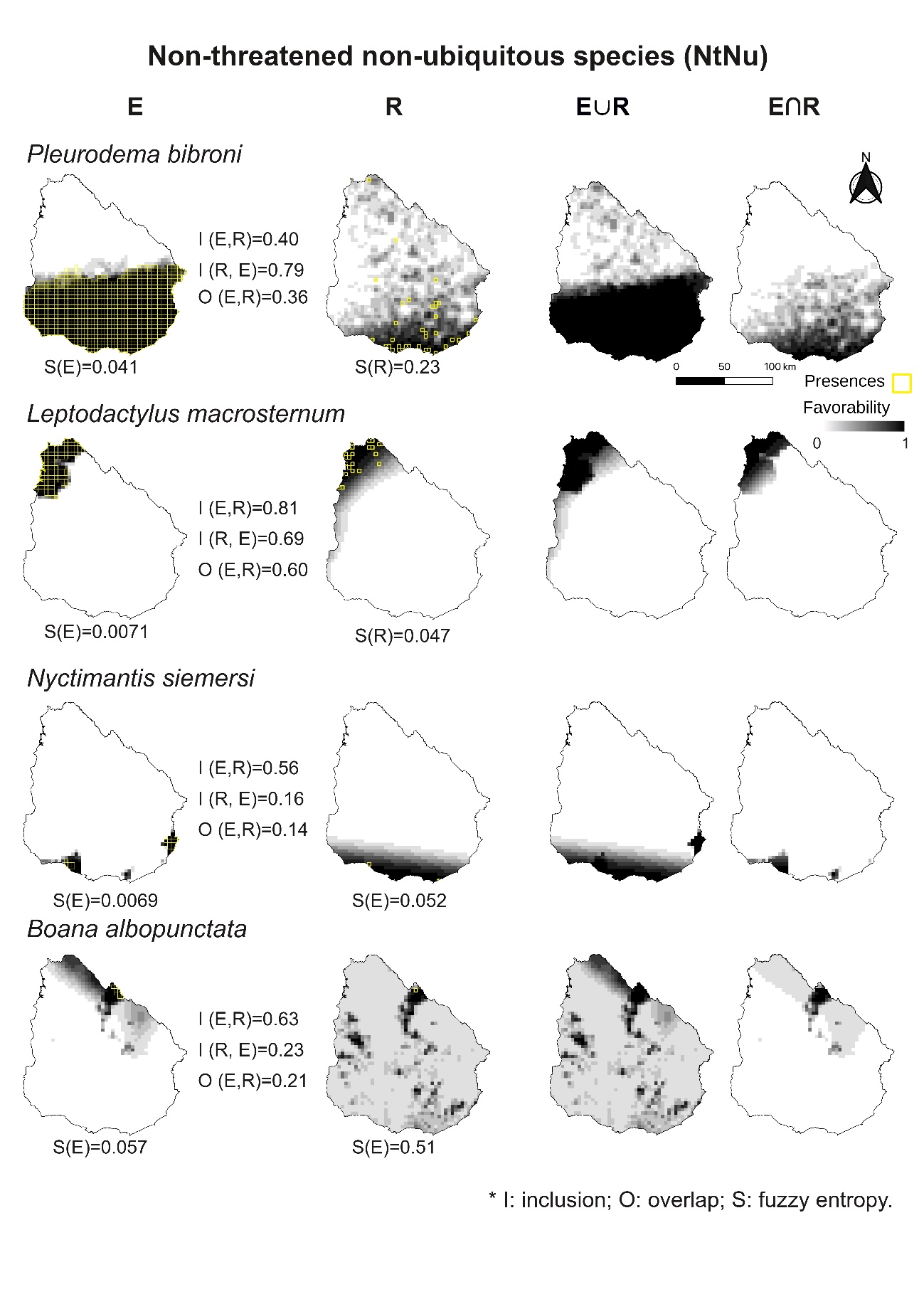
8


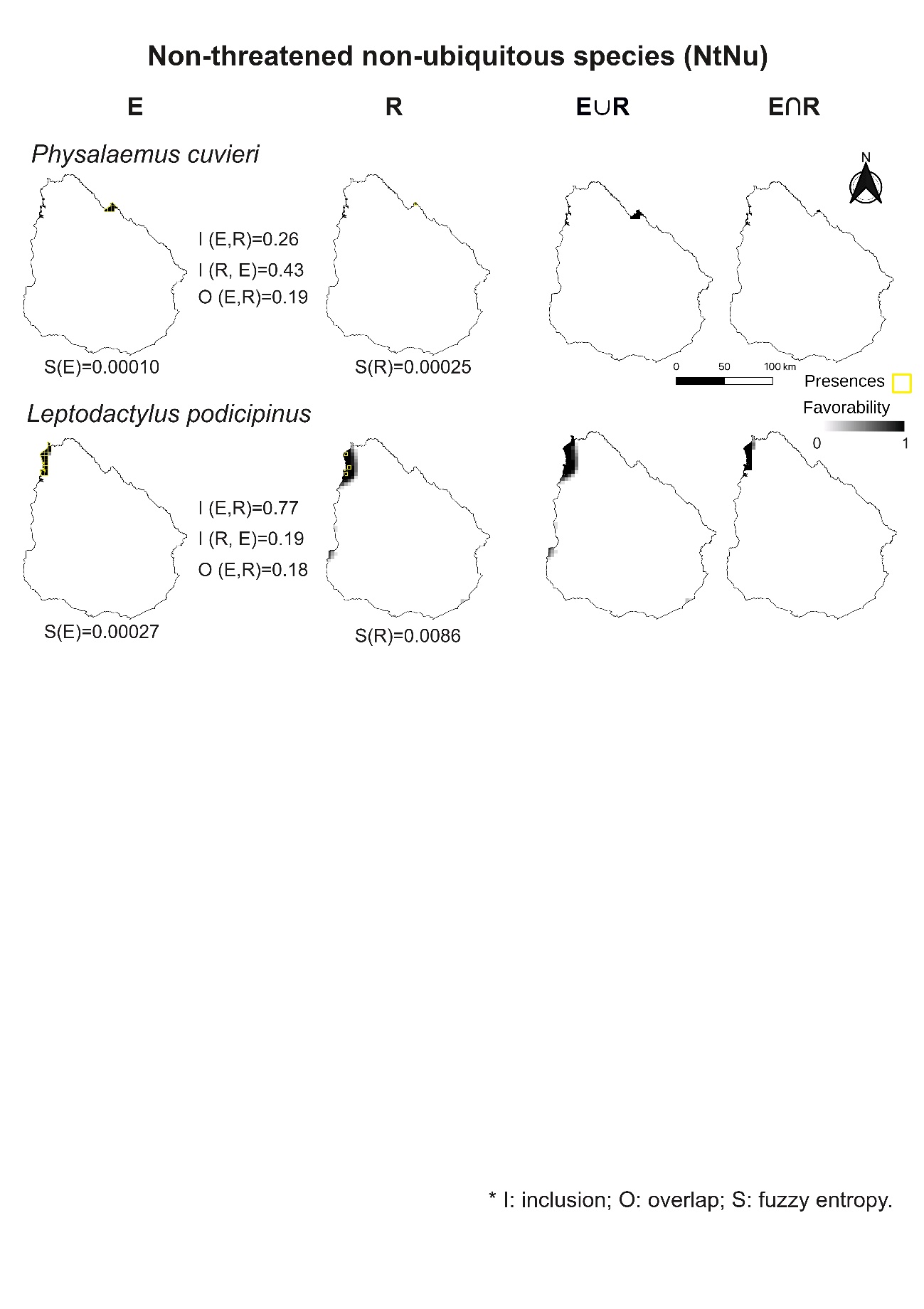


9


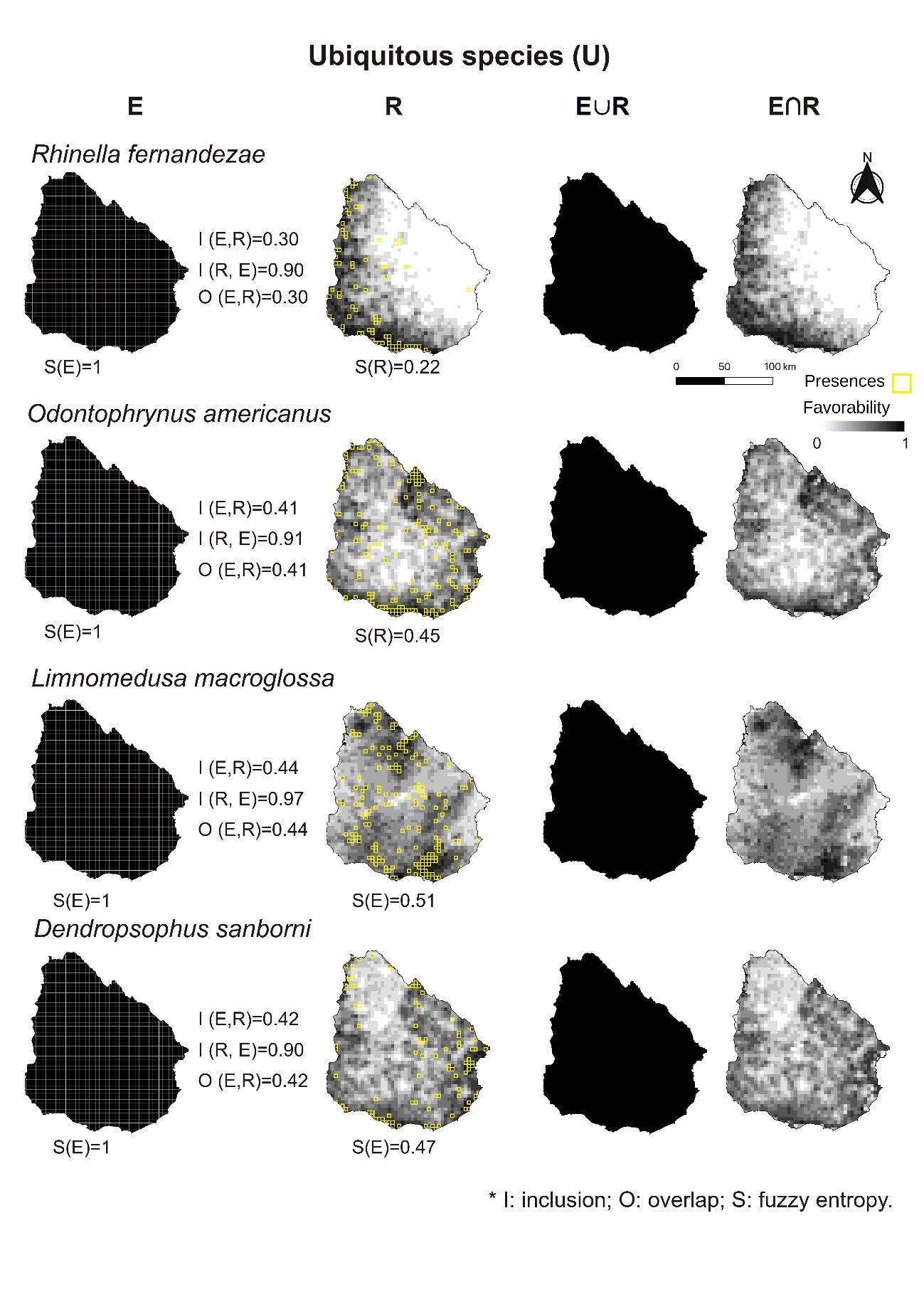


10


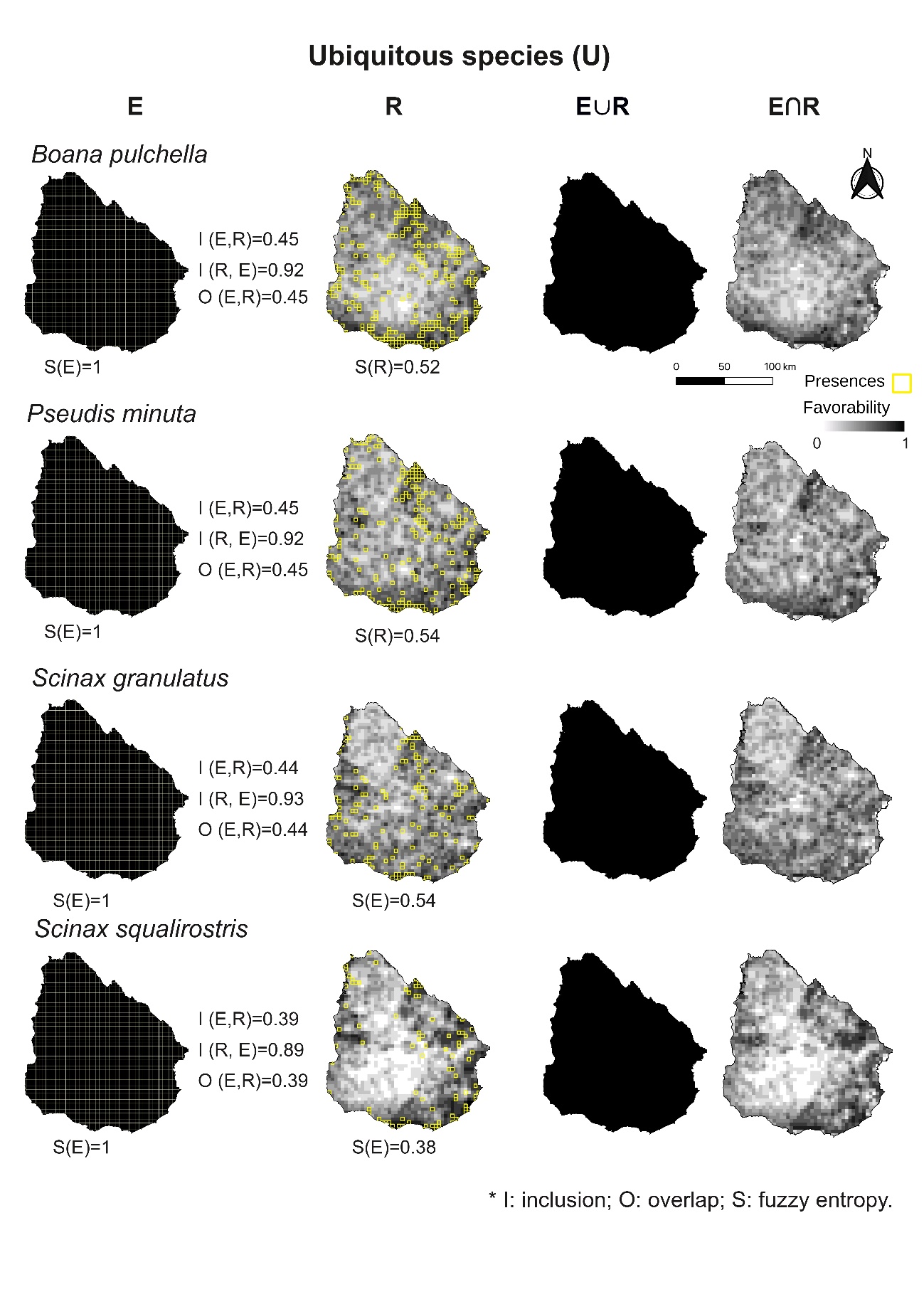
11


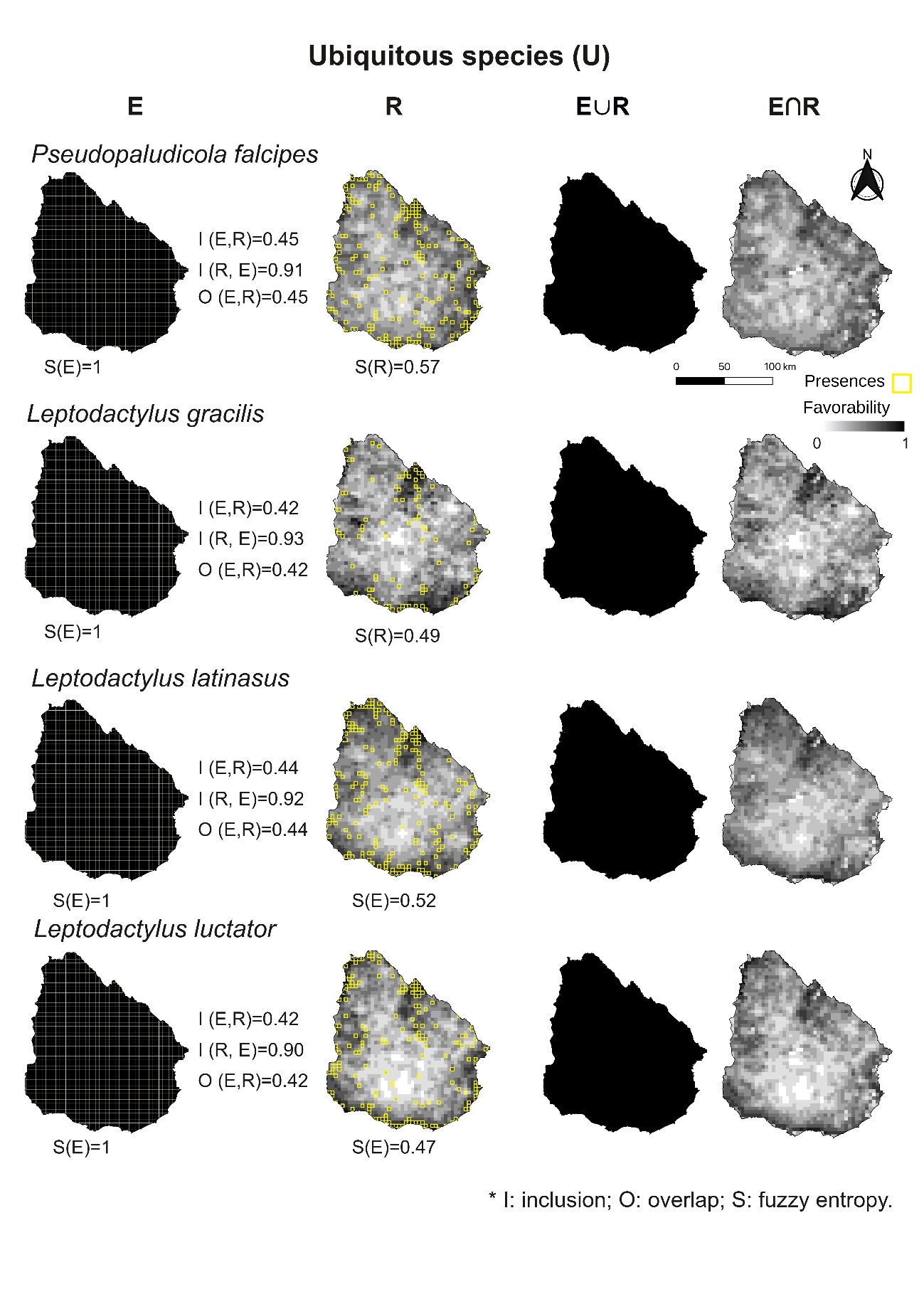


12

**
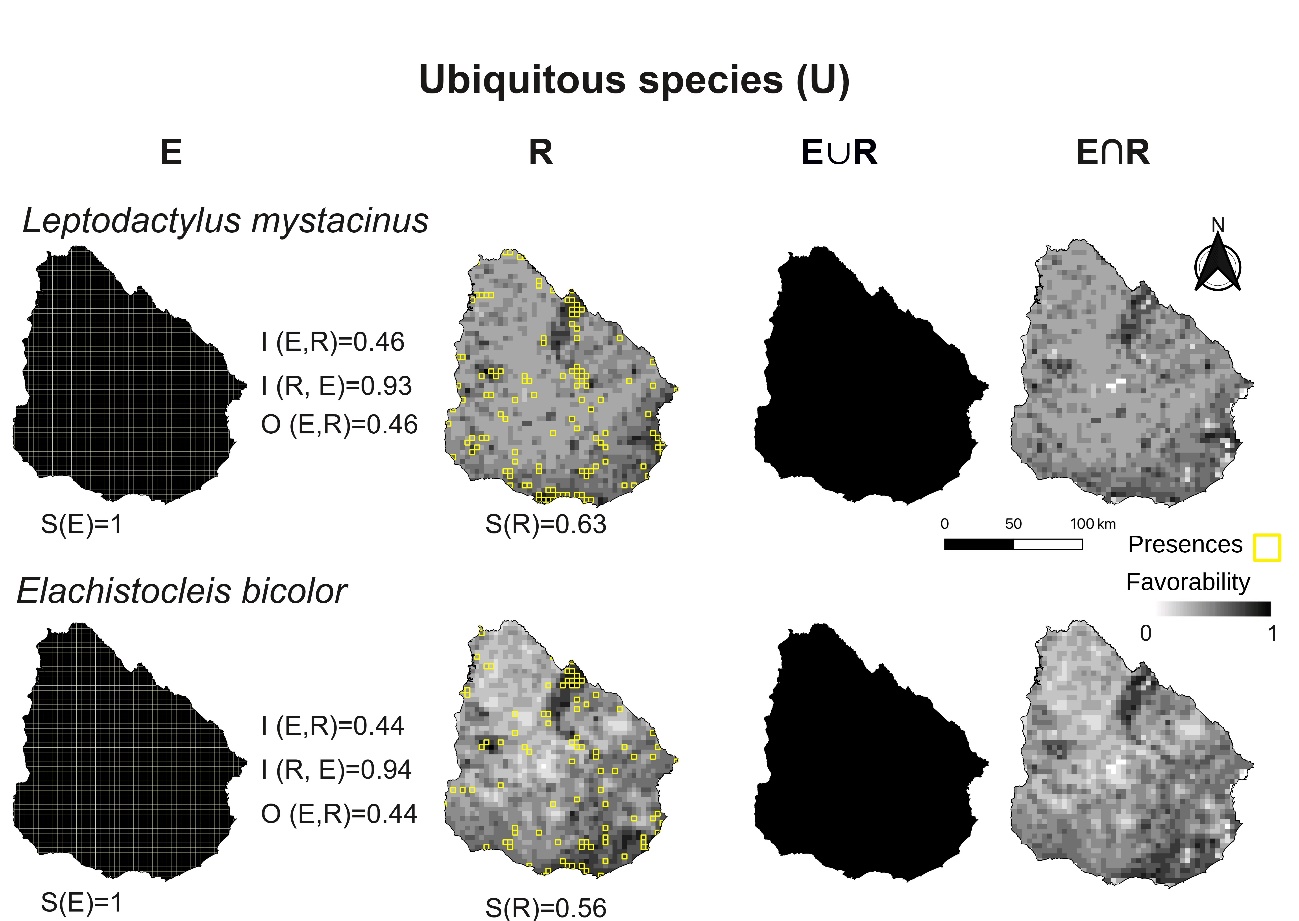
**

**Figure S3.** Favourability models for all amphibian species analyzed in Uruguay: threatened, non-threatened non-ubiquitous and ubiquitous species. We show from left to right the models based on expert knowledge (E), the models based on species records (R), and then their fuzzy union (E∪R) and fuzzy intersection (E∩R). The presences are shown on the models through the yellow border of the cell with presence. For the ubiquitous species the presences cover all of Uruguay according to the experts. I (E,R): degree of inclusion of the model based on expert criteria into the model based on species records. I (R,E): degree of inclusion of the model based on species records into the model based on expert criteria. O (E,R) degree of overlap between the models based on expert criteria and on species records. S(E): Entropy of the model based on expert criteria. S(R): Entropy of the model based on species records.

13
